# Effectiveness of nurse-led mHealth interventions on symptom outcomes in adult patients with cancer: a systematic review and meta-analysis

**DOI:** 10.1186/s12912-025-03981-2

**Published:** 2025-10-31

**Authors:** Chenxing Zhang, Yuezhen Hong, Yujia Chen, Rachel Arbing, Wei-Ti Chen, Feifei Huang

**Affiliations:** 1 School of Nursing, Fujian Medical University, No.1 Xueyuan Road, Minhou County, Fuzhou, Fujian 350122 China; 2 School of Nursing, University of California Los Angeles, 700 Tiverton Avenue, Los Angeles, CA 90095 USA

**Keywords:** Nursing intervention, Mobile health, Symptom management, Neoplasms, Meta-analysis, Systematic review

## Abstract

**Background:**

Patients with cancer face a high symptom burden, but traditional care models often lack timely, personalized support during home recovery. Nurse-led mobile health (mHealth) interventions show promise in bridging this gap. However, evidence on their effectiveness and reporting quality remains limited. This study evaluated the impact of nurse-led mHealth interventions on symptom outcomes in adult patients with cancer and assessed reporting completeness using the WHO mERA framework.

**Methods:**

A systematic search of 10 electronic databases included studies published before January 27, 2025. Eligible studies were randomized controlled trials (RCTs) evaluating nurse-led mHealth interventions for cancer symptom outcomes. The risk of bias was assessed via the Cochrane Risk of Bias 2.0 tool. The meta-analyses were conducted via random-effects models, with effect sizes expressed as standardized mean differences (SMDs) with 95% confidence intervals (CIs). Sensitivity analyses explored sources of heterogeneity. Intervention characteristics were categorized using the Omaha System, and reporting quality was evaluated with the mHealth Evidence Reporting and Assessment (mERA) checklist.

**Results:**

A total of 14 RCTs involving 1,972 participants were included. Pooled results from high-certainty evidence revealed that nurse-led mHealth interventions significantly improved depression (SMD = − 0.42, 95% *CI* [− 0.57, − 0.27], *p* < 0.001). There was low-certainty evidence suggesting potential benefits for quality of life (SMD = 0.49, 95% *CI* [0.17, 0.81], *p* = 0.003). Very low-certainty evidence indicated possible effects on symptom severity (SMD = − 0.49, 95% *CI* [− 0.81, − 0.17], *p* = 0.003) and anxiety (SMD = − 0.47, 95% *CI* [− 0.74, − 0.20], *p* < 0.001). No significant effects were observed for physical health, pain, or mental health. Interventions vary widely in platform, duration, and theoretical underpinnings. Most emphasized education and psychosocial support, with fewer studies targeting surveillance or treatment. mERA assessment revealed substantial reporting gaps, particularly regarding user feedback, cost, and data security.

**Conclusions:**

On the basis of high-certainty evidence, nurse-led mHealth interventions appear to benefit depression management. However, the evidence for their effects on quality of life, symptom severity, anxiety, and other outcomes is of low or very low certainty, limiting confidence in these findings. Moreover, variability in intervention design and inadequate reporting transparency may hinder broader adoption. Future research should prioritize theory-based, cancer specific, and multidisciplinary approaches while adhering to standardized reporting frameworks to support effective implementation and scalability.

**Registration:**

This systematic review was registered with the International Prospective Register of Systematic Reviews (PROSPERO); Registration number: CRD420251024506.

**Supplementary Information:**

The online version contains supplementary material available at 10.1186/s12912-025-03981-2.

## Introduction

Cancer remains a significant global health challenge, with an estimated 20 million new cases and 9.7 million deaths in 2022 [1]. The symptom burden of cancer and its treatments is widespread across all types, stages, and care phases [2], often leading to moderate-to-severe symptoms [3] that substantially impair patients’ functional status and quality of life (QOL) [2]. As such, effective symptom management is a core element of cancer care.

However, traditional follow-up models—such as in-person visits during clinic appointments or telephone calls—rely heavily on patient self-reports at the time of the follow-up visit. These approaches often provide limited real-time feedback from patients as they may not be able to recall or convey all symptoms during the scheduled visit, and do not allow for on-demand consultation which offers real-time access to a broader range of providers at any time, regardless of location. As a result, timely and personalized interventions are frequently delayed, particularly during treatment intervals (e.g., chemotherapy-free intervals or other inter-treatment periods), when patients are at home. Additionally, the growing number of patients with cancer further strain follow-up models that depend extensively on healthcare professionals for routine monitoring and support, adding to the workload and stress of these providers, which raises concerns about their continued sustainability. Moreover, reliance on in-person care may exacerbate health inequities, particularly for patients in rural or underserved areas, who face barriers to timely symptom assessment and follow-up [4, 5].

Mobile health (mHealth), as defined by the World Health Organization (WHO), involves the use of mobile and wireless technologies to support health goals and has emerged as a promising solution to the limitations of traditional care models. Over the past decade, global interest and investment have fueled the rapid growth of mHealth in both high- and low-resource settings [6]. While numerous randomized controlled trials (RCTs) have examined their effects on cancer symptom management, inconsistencies in intervention types, technology platforms, and outcome measures have resulted in mixed findings across systematic reviews, particularly for physical symptoms, psychological outcomes, and QOL [7–9]. Moreover, few existing reviews have neither specifically highlighted the pivotal role of nurses in multidisciplinary symptom management, nor have they evaluated the completeness and transparency of intervention reporting. Instead, prior reviews [9, 10] have focused primarily on the effectiveness of mHealth interventions.

Nurses play a central role in cancer care, with responsibilities spanning symptom assessment, patient education, care coordination, direct clinical care, symptom management, and the delivery of supportive interventions [11]. As oncology nurses are increasingly recognized as key providers of evidence-based symptom management across the cancer continuum [12], a growing number of RCTs have explored nurse-led mHealth interventions aimed at improving the symptom outcomes of patients with cancer. However, only two systematic reviews [13, 14] have focused specifically on this area—one limited to lung cancer with just four studies, and the other including quasi-experimental designs among patients receiving chemotherapy or radiotherapy. Both reviews were constrained by outdated studies and narrow outcome scopes (primarily pain and QOL).

In addition, the reporting quality of mHealth interventions varies considerably, hindering the comparison, interpretation, and replication of findings across studies. To address this, the WHO developed the mHealth Evidence Reporting and Assessment (mERA) checklist [6], which specifies the minimum amount of information needed to ensure clear and reproducible reporting of mHealth interventions. Despite its potential to enhance transparency and consistency, the mERA checklist has not yet been applied in reviews assessing nurse-led mHealth interventions in cancer care.

Given the limitations of existing reviews—such as narrow patient populations, outdated evidence, limited outcome scope, and lack of reporting on quality assessments—there is a clear need for a comprehensive and updated systematic review and meta-analysis. This review aims to (1) characterize the features of existing nurse-led mHealth interventions for symptom outcomes in patients with cancer, (2) synthesize evidence from RCTs evaluating the effectiveness of nurse-led mHealth interventions for symptom outcomes in patients with cancer, and (3) apply the mERA checklist to assess the reporting quality of included studies, offering methodological guidance to inform future research and clinical practice.

## Methods

### Protocol and registration

This systematic review was registered with the International Prospective Register of Systematic Reviews (PROSPERO; registration number: CRD420251024506).

### Search strategy

To identify eligible studies, we conducted a comprehensive search across 10 electronic databases, including five English-language databases (PubMed, Web of Science, CINAHL, the Cochrane Library, and Embase) and five Chinese databases (CNKI, WanFang, VIP, SinoMed, and the Chinese Medical Journal Database). A full search strategy was applied to each database, with detailed search strings provided in Additional file 1: Table S1. The search covered all records from database inception to January 27, 2025, with no filters or restrictions applied. Additionally, we performed a backward search of reference lists from all included studies. Eligibility criteria were based on the PICOS framework (Population, Intervention, Comparison, Outcomes, and Study design), as outlined in Table 1.

**Table 1:** Inclusion and exclusion criteria

	Inclusion criteria	Exclusion criteria
P (population)	Participants who are diagnosed with cancer and are at least 18 years old.	Commentary, gray literature, duplicate article, conference abstract, letter, review, protocol, and editorial; study that combines interventions of mHealth and other approaches where data for the mHealth component cannot be extracted separately; and study with incomplete mHealth-related data, despite attempts to contact the authors for supplementary information.
I (intervention)	The study examines nurse-led mHealth interventions for managing cancer-related physical and psychological symptoms. A nurse-led mHealth intervention refers to a healthcare approach led by nurses that delivers nursing care and supports symptom management via mobile devices or wireless communication technologies. These mHealth interventions encompass platforms such as WeChat, mini-programs, mobile applications, websites, smartphones, wearable devices, and portable virtual reality (VR) systems.	
C (comparison)	Any comparator was acceptable, including wait-list control groups, non-intervention groups (care-as-usual), and non-mHealth interventions.	
O (outcomes)	The primary outcomes are physical or psychological symptoms and quality of life.	
S (study design)	Randomized controlled trials reported in English or Chinese.	

### Study selection

Two independent reviewers screened titles and abstracts on the basis of predefined eligibility criteria and excluded irrelevant studies. The full-text articles deemed potentially relevant were then reviewed for final inclusion. Discrepancies were resolved through discussion with a third reviewer.

### Data extraction

Data extraction was conducted by a primary reviewer via predefined tables and verified by a second reviewer. The extracted data included study characteristics (e.g., first author, publication year, country, design, and setting), participant details (e.g., sample size, mean age, gender, cancer type, and follow-up duration), intervention features (e.g., aim, theoretical framework, mHealth technology type, components, dose/frequency/duration, and provider), and relevant outcomes (e.g., severity, frequency, and distress of physical or psychological symptoms). For studies with missing or incomplete outcome data, the corresponding authors were contacted. If no response was received or data remained unavailable, the study was included in the qualitative synthesis only or excluded from the quantitative analysis as appropriate.

### Quality assessment

The methodological quality of the included RCTs was independently assessed via the Cochrane Risk of Bias tool 2.0 (RoB 2.0) [15], which evaluates five domains: the randomization process, deviations from intended interventions, missing outcome data, outcome measurement, and selection of the reported result. Each domain was rated as low risk, some concerns, or high risk, with the overall risk determined by the highest domain rating [15].

The certainty of evidence was evaluated via the GRADE framework, which categorizes evidence as high, moderate, low, or very low on the basis of risk of bias, inconsistency, imprecision, indirectness, and publication bias [16].

Furthermore, the quality of the mHealth evidence reported in the included studies was assessed via the mERA checklist. This tool comprises 16 items covering key domains such as population-level infrastructure, the technology platform, interoperability and health information systems context, intervention delivery and content, usability and content testing, user feedback, participant access, cost assessment, program adoption and entry, scalability limitations, contextual adaptability, replicability, data security, regulatory compliance, and intervention fidelity.

### Data synthesis and analysis

Before pooling the effect sizes, the outcome directions were standardized across studies to ensure consistent interpretation. For the outcomes assessed via different instruments (e.g., depression measured by the PHQ-9 or HADS-D), standardized mean differences (SMDs) with 95% confidence intervals (CIs) were calculated to allow pooling on a common scale. For measures where higher scores indicated better outcomes, the sign of the effect size was reversed. Sensitivity analyses were conducted as appropriate to assess the robustness of the pooled estimates. A *p*-value of < 0.05 was considered statistically significant.

When studies reported outcomes at multiple time points, we extracted data from the longest follow-up to avoid multiplicity and to capture the sustained effects of the intervention, following the recommendations of the Cochrane Handbook for Systematic Reviews of Interventions [15].

For synthesis, the included studies were grouped according to outcome type. These outcomes were extracted based on the data reported in the publication. The outcomes were categorized into three major domains: (1) psychological outcomes, including depression, anxiety, and mental health; “mental health” refers to overall psychological well-being, as assessed by multidimensional instruments such as the FACIT-SP or the SF-36 mental health domain. (2) Physical outcomes, including symptom severity, physical health, pain, fatigue, lymphedema symptoms, urinary continence and sexual function; “physical health” refers to overall or general physical well-being, as measured by multidimensional instruments such as the SF-12 or FACT-B. (3) Quality of life.

Meta-analyses and heterogeneity assessments were conducted via Review Manager Web (RevMan Web, version 9.10.0). For outcomes that could not be quantitatively synthesized due to insufficient data or a lack of basic statistics (e.g., means and standard deviations), a narrative synthesis was performed to describe the intervention effects. Differences in measurement tools were addressed via SMDs. SMDs (Hedges’ g) were generated using RevMan Web to compare nurse-led mHealth interventions with usual care, standard care, or waitlist controls. Hedges’ g was applied with a small-sample correction, ensuring direct harmonization across different outcome measures. In the single three-arm trial, only the usual care control group was included as a comparator, whereas the active control arm (“friendly” visit) was excluded to maintain consistency with the review question.

Heterogeneity was assessed via the *χ*^*2*^ test (*p* < 0.05 indicated statistical significance) and the *I²* statistic (25%, 50%, and 75% representing low, moderate, and high heterogeneity, respectively) [17]. In accordance with the latest Cochrane Handbook recommendations [15], we applied a random-effects model in all meta-analyses, as variations in clinical interventions are expected across trials. The *I²* statistic was reported to quantify the degree of heterogeneity but was not used to determine the choice of effect model. The*τ²* was estimated via the DerSimonian-Laird method, and CIs were calculated using the Wald-type method. A priori, we determined that a study would be considered influential if its exclusion led to a substantial reduction in heterogeneity *(I²*). In such cases, we conducted sensitivity analyses by comparing the pooled results with and without the study. Formal tests for publication bias (e.g., Egger’s test) were not conducted because fewer than ten studies contributed to each outcome, which would render such tests underpowered and potentially misleading [15].

### Classification of interventions

Interventions were categorized according to the Omaha System nursing intervention classification, which classifies nursing interventions into four categories: teaching, guidance, and counseling; treatments and procedures; case management; and surveillance [18]. To enhance applicability in oncology nursing, we also incorporated supplementary descriptions provided by the Recognising European Cancer Nursing (RECaN) project [19], which offered additional clarifications of intervention domains in cancer care contexts. For detailed classification criteria, refer to the study by Charalambous et al. [19].

## Results

### Study selection

**Fig. 1:**
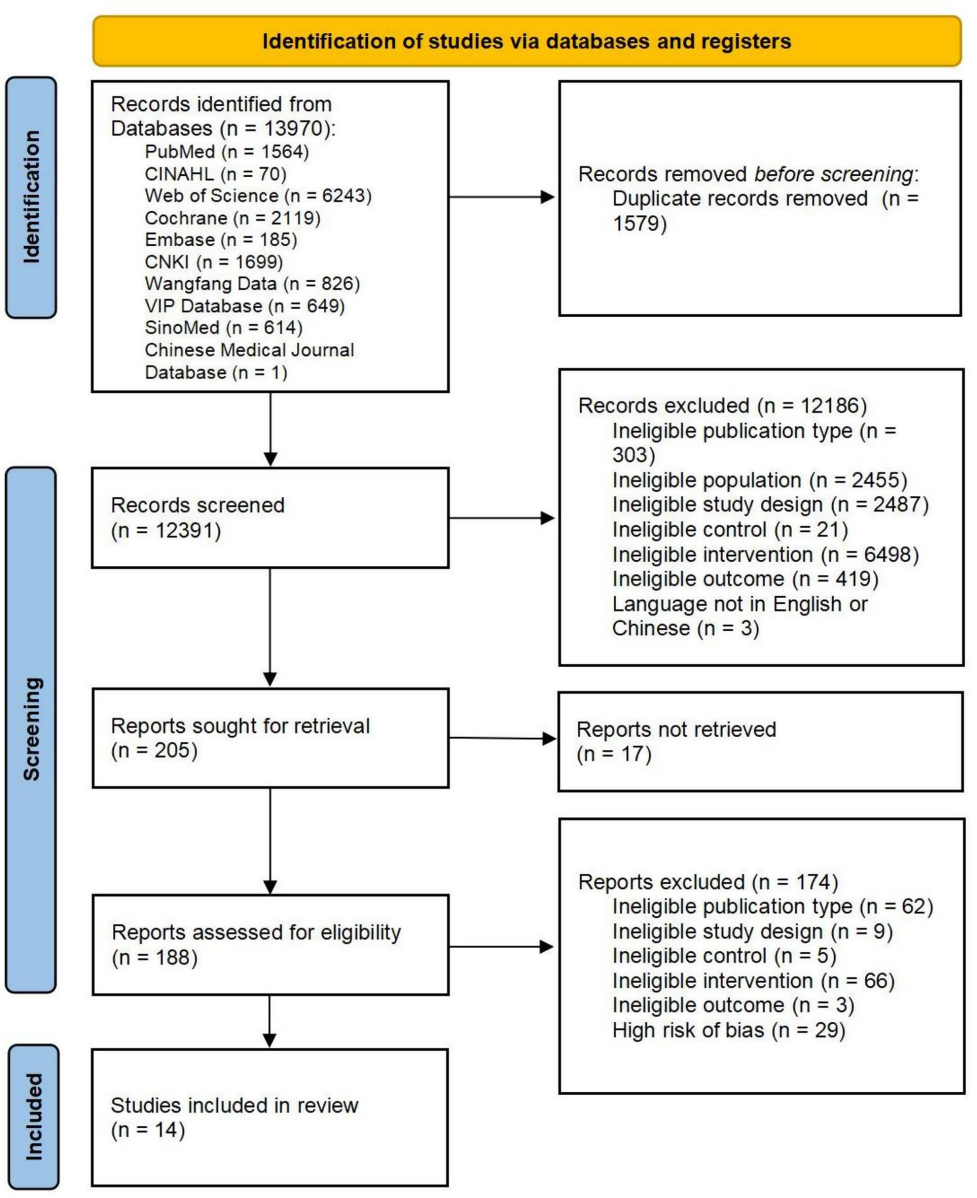
PRISMA flow chart of study selection process for the meta-analysis Abbreviations: CINAHL, Cumulative Index to Nursing and Allied Health Literature; CNKI, Chinese National Knowledge Infrastructure; VIP database, China Science and Technology Journal Database

Figure 1 shows the PRISMA 2020 flow diagram. From the 13,970 records identified, 12,391 remained after duplicate removal and were screened by title and abstract. Among the 205 full-text articles assessed, 43 met the initial criteria, but 29 were excluded because of a high risk of bias. Fourteen studies with low to moderate risk were included in the review.

### Risk of bias of studies

Overall, among the 14 included RCTs, 6 studies were judged to have a low risk of bias, and 8 studies had some concerns. Among the 14 included RCTs, five [19–23] did not blind participants or investigators. One study [24] conducted a per-protocol analysis, excluding participants who did not receive the assigned intervention. Another study [25] did not report allocation concealment, and one reported significant baseline differences between the control and intervention groups [26]. (Additional file 2: Figure S1 and Figure S2)

### Study characteristics

A total of 14 RCTs published between 2010 and 2025 involving 1,972 adult patients with cancer were included. Studies were conducted in China (*n* = 6) [21, 22, 26–29], the United States (*n* = 3) [19, 30, 31], Egypt (*n* = 1) [23], Sweden (*n* = 2) [12, 19], Iran (*n* = 1) [25], and South Korea (*n* = 1) [4]. One study [31] covered both urban and rural settings, while most were based in urban hospitals, healthcare centers, or clinics. The sample sizes ranged from 31 to 405 participants. Breast cancer was the most common diagnosis (*n* = 6) [23, 25–27, 30], followed by ovarian [19], cervical [21], lung [21], colorectal [32], digestive system [29], prostate [24], gastric [28], and mixed cancers [31], each represented by a single study. Intervention timing varied across studies: six focused on patients in the recovery or survivorship phase [21, 22, 24, 26, 27, 30], five focused on those undergoing active treatment [20, 23, 28, 31, 32], and three included participants at mixed or unspecified stages [19, 25, 29]. Further details are provided in Table 2.

**Table 2:** Study characteristics

No.	Author, year, study country	Study design	Setting	Sample			Cancer type	Intervention group (IG)	Control group (CG)	Follow-up	
				Sample size	Age in years mean/median (SD/IQR)	Gender *n* (%)				Duration	Withdrawls
1	Kroenke K et al., 2010, USA	Two-arm RCT	Community-based urban and rural oncology practices	Total: 405 IG: *n* = 202; CG: *n* = 203	IG: 58.7 (11.0); CG: 59.0 (10.6)	Female: IG: 128 (63); CG: 147 (72)	Mixed	Centralized telecare management by nurse-physician specialist team with automated home-based symptom monitoring by interactive voice recording or internetInternet	Usual care	Baseline 1 month 3 months 6 months 12 months	Total: 136 IG: *n* = 68; CG: *n* = 68
2	Donovan HS et al., 2014, USA	Two arm RCT	NR	Total: 65 IG: *n* = 33; CG: *n* = 32	IG: 55.94 (10.37); CG: 57.12 (2.31)	Female: IG: 33 (100); CG: 32 (100)	Ovarian	WRITE (Written Representational Intervention to Ease Symptoms)	Wait-List	Baseline 13 weeks 17 weeks	Total: 10; IG: *n* = 9; CG: *n* = 1
3	Ghanbari E et al., 2021, Iran	Two-arm RCT	Breast health clinics	Total: 82 IG: *n* = 41; CG: *n* = 41	IG: 46.9 (9.83); CG: 46 (8.80)	Female: IG: 41 (100); CG: 41 (100)	Breast	BCSzone app	Wait-List	Baseline 5 weeks	Total: 5 IG: *n* = 3; CG: *n* = 2
4	Fu MR et al., 2022, USA	Two-arm RCT	Academic cancer center	Total: 120 IG: *n* = 60; CG: *n* = 60	IG: 56.6 (10.3); CG: 56.8 (11.0)	Female: IG: 60 (100); CG: 60 (100)	Breast	The-Optimal-Lymph-Flow (TOLF) program	Arm Precaution (AP) program	Baseline 12 weeks	Total: 6; IG: *n* = 5; CG: *n* = 1
5	Hao Q et al., 2024, China	Two-arm RCT	Hospitals	Total: 184 IG: *n* = 92; CG: *n* = 92	IG: 52.9 (10.5); CG: 51.0 (10.5)	Female: IG: 92 (100); CG: 92 (100)	Cervical	WeChat-based cognitive behavioural stress management (WB-CBSM)	Usual care	Baseline 1 month 3 months 6 months	Total: 14; IG: *n* = 6; CG: *n* = 8
6	Hwang YJ et al., 2025, South Korea	Two-arm RCT	900-bed hospital	Total: 31 IG: *n* = 15; CG: *n* = 16	IG: 62.18 (7.18); CG: 63.29 (7.10)	Female: IG: 11 (64.7); CG: 10 (58.8)	Colorectal	App-based physical activity program with Information-Motivation-Behavioral (IMB) skills model	Standard booklet education on exercise and neuropathy management	Baseline 4 weeks 6 weeks	Total: 3; IG: *n* = 2; CG: *n* = 1
7	Sui Y et al., 2020, China	Two-arm RCT	Academic hospital	Total: 200 IG: *n* = 100; CG: *n* = 100	IG: 61.37 (11.21); CG: 62.35 (9.98)	Male: IG: 80 (80); CG: 84 (84)	Lung	WeChat-based education and rehabilitation program (WERP)	Conventional face-to-face education and telephone/clinic follow-up	Baseline 3 months 6 months 9 months 12 months	Total: 30; IG: *n* = 10; CG: *n* = 20
8	Tawfik E et al., 2023, Egypt	Three-arm RCT	Chemotherapy unit within oncology center	Total: 150 IG: *n* = 50; CG1 : *n* = 50; CG2: *n* = 50	IG: 45.68 (8.49); CG1: 43.84 (5.90); CG2: 45.38 (7.48)	Female: IG: 50 (100); CG1: 50 (100); CG2: 50 (100)	Breast	ChemoFreeBot chatbot providing personalized education	CG1: Nurse-led education with three face-to-face sessions. CG2: General information during chemotherapy	Baseline 4 weeks	Total: 0; IG: *n* = 0; CG: *n* = 0
9	Wang L et al., 2022, China	Two-arm RCT	Breast cancer ward of tertiary hospital	Total: 103 IG: *n* = 51; CG: *n* = 52	IG: 45.37 (7.59); CG: 48.17 (8.05)	Female: IG: 51 (100); CG: 52 (100)	Breast	4-week internet-delivered mindfulness-based cancer recovery (iMBCR) program	Usual care plus health education sessions (non-mindfulness)	Baseline 1 month	Total: 3; IG: *n* = 1; CG: *n* = 2
10	Zheng M et al., 2022, China	Three-arm RCT	Oncology department of academic general hospital	Total: 150 IG: *n* = 50; CG1: *n* = 50; CG2: *n* = 50	IG: 57.48 (9.29); CG1: 59.50 (10.67); CG2: 58.46 (9.96)	Male: IG: 43 (35); CG1: 41 (33); CG2: 39 (38)	Digestive system	WeChat-based life review program	CG1: Friendly visits via WeChat + routine care; CG2: Routine care only	Baseline 2 days 1 month 6 months	Total: 15; LRG: *n* = 4; CG1: *n* = 6; CG2: *n* = 5
11	Du X et al., 2025, China	Two-arm RCT	Breast surgery ward of general tertiary level academic hospital	Total: 86 IG: *n* = 45; CG: *n* = 41	NR	NR	Breast	The-Optimal-Lymph-Flow (TOLF) health IT system	Routine treatments and nursing care	Baseline 1 month 3 months	Total: 18; IG: *n* = 7; CG: *n* = 11
12	Wennerberg C et al., 2023, Sweden	Two-arm RCT	Urology departments at county hospitals	Total: 165 IG: *n* = 83; CG: *n* = 82	IG: 64 (6.2); CG: 64 (6.3)	Male: IG: 83 (100); CG: 82(100)	Prostate	Electronic Patient Activation in Treatment at Home (ePATH)	Standard care	Baseline 1 month 3 months 6 months 12 months	Total: 24; IG: *n* = 13 CG: *n* = 11
13	Zhang S et al., 2025, China	Two-arm RCT	General tertiary-level hospital	Total: 82 IG: *n* = 41; CG: *n* = 41	IG: 52.29 (8.66); CG: 53.59 (8.69)	Male: IG: 21 (51.2); CG: 16 (39.0)	Gastric	Cancer-Related Fatigue Management Program	Routine care	Baseline 3 months 4 months	Total: 10; IG: *n* = 5 CG: *n* = 5
14	Fjell M et al., 2020, Sweden	Two-arm RCT	Outpatient oncology departments in two academic hospitals	Total: 149 IG: *n* = 74; CG: *n* = 75	IG: 48 (10.6); CG: 50 (11.6)	Female: IG: 74 (100); CG: 75 (100)	Breast	Interaktor app	Standard care	Baseline 20 weeks	Total: 9; IG: *n* = 5 CG: *n* = 4

Note: Follow-up durations reported in this table refer to the time elapsed from randomization

**Abbreviations**: IG, intervention group; CG, control group; NR, not reported; RCT, randomized controlled trial; CIPN, chemotherapy-induced peripheral neuropathy

### Characteristics of the nurse-led mHealth interventions

The characteristics of the nurse-led mHealth interventions are summarized in Table 3.

**Table 3:** Intervention summary and study outcomes

Study	Intervention aim	Theoretical framework	mHealth type	Intervention components	Patient phase during intervention delivery	Intervention dosage (frequency/duration)	Intervener	Effectiveness primary outcomes [mean (SD)]	Effectiveness secondary outcomes [mean (SD)]
Kroenke K et al., 2010, USA	Determine whether centralized telephone-based care management with automated symptom monitoring can improve depression and pain in patients with cancer.	NR	Telephone and web	• Care management • Automated symptom monitoring • Medication management	Undergoing treatment	• Telephonic care management: Baseline; 1-week; 4-weeks; 12-weeks; Special circumstances/ NR • Monitoring survey: twice a week for the first 3 weeks; weekly during weeks 4–11; twice a month during months 3–6; once a month during months 7–12/NR	Trained nurse care manager and participant’s oncologist	• Depression severity (HSCL-20) [IG: 1.06 (0.65) & CG:1.32 (0.83)] (*p* < 0.001) • Pain severity (BPI) [IG: 3.62 (2.42) & CG: 4.33 (2.21)] (*p* < 0.001)	• Physical health (SF-12) [IG: 35.1 (10.2) & CG: 35.6 (10.7)] (*p =* 0.52) • Mental health (SF-12) [IG: 46.6 (12.3) & CG: 43.3 (12.6)] (*p =* 0.012) • Anxiety severity (GAD-7) [IG: 5.82 (5.57) & CG: 6.48 (6.16)] (*p =* 0.002) • QOL (Single-item 0–10 scale) [IG: 6.20 (2.27) & CG: 6.07 (2.18)] • Symptom burden (22-item somatic symptom scale) [IG: 15.9 (7.0) & CG: 16.9 (7.3)]
Donovan HS et al., 2014, the USA	Evaluate feasibility, usability, satisfaction, and initial efficacy of WRITE Symptoms for improving symptom management in women with recurrent ovarian cancer.	RA	Web	• Representational assessment • Identifying and exploring gaps, errors, and confusions • Creating conditions for conceptual change • Introducing replacement information • Summary • Goal setting and planning • Follow-up contact: goal and strategy review	Mixed or unspecified	NR/NR	Master’s prepared oncology nurses trained in RA	• Symptom severity (SQR) [IG: 4.58 (3.33) & CG:4.80 (1.53)] (*p* = 0.626)	-
Ghanbari E et al., 2021, Iran	Effects of psychoeducational interventions on anxiety and self-esteem in women with breast cancer using mobile app and mobile-based online group discussions.	NR	App	• Breast cancer related knowledge education • Stress management • Promoting self-esteem • Anger management	Mixed or unspecified	NR/4 weeks	Psychiatric nurse	• Anxiety (STAI) [IG: 90.66 (13.84) & CG: 106.92 (15.94)] (*p <* 0.001)	-
Fu MR et al., 2022, USA	Evaluate effectiveness of web- and mobile-based TOLF systems for managing chronic pain and symptoms related to lymphedema in breast cancer survivors.	Model of Self-Care for Lymphedema Symptom Management	Web- and mobile-based platform	• Health care education (including lymphedema knowledge, healthy weight, and self-care strategies modules) • Exercise (daily lymphatic exercises module) • ICounseling (information consultation module)	Recovery or survivorship	• At least twice a day/12 weeks	Nurse	• General bodily pain (BCLE-SEI) [IG: 1 (0-1.5) & CG: 1 (1–3)] (*p* = 0.04)	• QOL (PIQ-6) [IG: 48.4 (7.9) & CG: 50.7 (8.1)] (*p* = 0.13) • Number of lymphedema symptoms (PIQ-6) [IG: 6.1 (5.1) & CG: 7.6 (5.2)] (*p* = 0.11)
Hao Q et al., 2024, China	Investigate effects of WB-CBSM on mental health, spiritual well-being, and QOL in patients with early-stage cervical cancer treated with surgical resection.	CBSM	WeChat	• Professional knowledge learning • One-on-one rehab training • One-to-one communication with nurses • WeChat group Communication	Recovery or survivorship	• NR/8 weeks	Nurse	• Anxiety (HADS-A) [IG: 6.0 (2.2) & CG: 7.0 (2.5)] (*p* = 0.007)	• Depression (HADS-D) [IG: 6.3 (2.3) & CG: 7.2 (2.3)] (*p* = 0.015) • Mental health (FACIT-SP) [IG: 39.2(4.7) & CG: 36.7 (4.6)] (*p =* 0.001) • QOL (QLQ-C30-global health status) [IG: 81.3 (14.7) & CG: 75.8 (14.6)] (*p* = 0.016) • Symptoms (QLQ-C30-Symptoms) [IG: 23.8 (13.4) & CG: 27.7 (14.2)] (*p* = 0.065)
Hwang YJ et al., 2025, South Korea	Examine effects of app-based physical activity program on alleviating peripheral neuropathy symptoms in patients with colorectal cancer.	IMB model	App	• Information provision (CIPN symptoms and exercise management) • Exercise goal setting with reminders • Automated exercise time monitoring (via app) • Exercise videos (aerobic, strength, balance) • Q&A function for real-time support	Undergoing chemotherapy	• NR/6 weeks	Master’s prepared nurse with over 10 years of experience in surgery department	• Peripheral neuropathy symptoms: [IG: 24.00 (12.07) & CG: 29.50 (14.02)] *p =* 0.004 • QOL [(IG: 10.93 (1.52) & CG: 8.88 (2.15)] *p =* 0.014	-
Sui Y et al., 2020, China	Investigate effects of WERP on anxiety, depression, QOL, loss to follow-up, and survival in NSCLC patients post-surgical resection.	NR	App	• Disease-related health education • Rehabilitation exercise guidance • Daily activity supervision • Psychological support	Recovery or survivorship	• Disease-related health education: Weekly video courses/12 weeks • Rehabilitation exercise guidance: Weekly video courses/40 weeks (from week 13 to week 52) • Daily activity supervision: Weekly step-counting supervision via WeChat Movement/12 months • Psychological support: Biweekly video counseling/12 months	Trained nurses with one month special training related to research content	• Anxiety (HADS-A) [IG: 5.00 (2.84) & CG: 6.69 (4.01)] (*p =* 0.001) • Depression (HADS-D) [IG: 5.22 (2.77) & CG: 6.55 (3.42)] (*p =* 0.003) • QOL (QLQ-C30-global health status) [IG: 74.44 (12.06) & CG: 70.26 (17.29)] (*p* = 0.049) • Symptoms (QLQ-C30-Symptoms) [IG: 27.29 (11.72) & CG: 27.86 (12.69)] (*p* = 0.742)	-
Tawfik E et al., 2023, Egypt	Compare effects of ChemoFreeBot, nurse-led education, and routine care on improving self-care behaviors and reducing chemotherapy-related side effects in women with breast cancer.	Empowerment Education Model	Chatbot	• Personalized education • Automated symptom monitoring • On-demand information access	Undergoing treatment	• Unlimited access/From first day of chemotherapy until post-intervention assessment	Nurse	• Physical symptoms (MSAS) [IG: 1.43 (0.29) & CG: 2.18 (0.47)] (*p <* 0.001) • Psychological symptoms (MSAS) [IG: 1.40 (0.43) & CG: 2.06 (0.42)] (*p <* 0.001) • Symptom burden (MSAS) [IG: 1.42 (0.30) & CG: 2.14 (0.39)] (*p* < 0.001)	-
Wang L et al., 2022, China	Investigate effectiveness of 4-week iMBCR program in reducing symptom burden and enhancing HRQOL in patients with breast cancer.	Heuristic framework	WeChat	• Weekly online mindfulness sessions (1.5 h/week) • Daily home mindfulness practices (30 min/day) • Educational materials (MBCR book, guided audio recordings) • Group discussions and participative sessions • Therapist-led sessions with mindfulness-trained professionals	Recovery or survivorship	• Weekly online sessions (4 sessions over 4 weeks)/4 weeks for online sessions; daily home practice for 28 days	Therapist with mindfulness training and 4 years of experience in teaching MBCR; Assistant with 2 years of experience in mindfulness practice	• Symptom burden (MDASI-C) [IG: 39.14 (9.95) & CG: 49.13 (10.43)] (*p* < 0.001) • QOL (FACT-B) [IG: 98.00 (7.51) & CG: 86.52 (10.88)] (*p <* 0.001) • Physical well-being (FACT-B) [IG: 22.88 (2.02) & CG: 20.86 (4.76)] (*p =* 0.002) • Emotional well-being (FACT-B) [IG: 18.46 (1.95) & CG: 17.26 (3.59)] (*p =* 0.42)	-
Zheng M et al., 2022, China	Examine effects of WeChat-based life review program on psychospiritual well-being of patients with digestive system cancer.	Life Review Theory	WeChat	• Synchronous e-life review interviews (40–60 min weekly • Asynchronous modules: Memory prompts, review extraction, mind space, E-legacy products	Mixed or unspecified	• Weekly sessions for 4 weeks/40–60 min	Registered nurse with more than 25 years of experience in clinical cancer care and 50 h of life review training	• Anxiety (HADS-A) [IG: 2.98 (2.71) & CG: 4.50 (3.54)] (*p =* 0.01) Depression (HADS-D) [IG :2.80 (1.91) & CG: 4.88 (4.51)] (*p =* 0.004)	-
Du X et al., 2025, China	Evaluate application effects of TOLF IT System in patients at high risk of developing breast cancer-related lymphedema.	Model of Self-Care for Lymphedema Symptom Management	Web and mobile platform	• Health care education (lymphedema knowledge, healthy weight, and self-care strategies modules) • Exercise (daily lymphatic exercises module) • ICounseling (information consultation module)	Recovery or survivorship	• NR/3 months	Nurse	• Physical health (SF-36) [IG: 29.49 (12.64) & CG: 34.24 (13.20)] (*p =* 0.256) • Mental health (SF-36) [IG: 47.62 (16.61) & CG: 47.36 (11.50)] (*p* = 0.931) • Symptom severity (BCLE-SEI) [IG: 5.00 (4.59) & CG: 12.18 (6.53)] (*p* < 0.001)	-
Wennerberg C et al., 2023, Sweden	Investigate effects of ePATH intervention on (1) urinary continence and (2) sexual function and adherence to self-care recommendations in (3) pelvic floor muscle exercises and (4) physical activity.	Self-determination theory	Web and mobile app	• Self-care support • Health information provision • Messaging function • Supplementary support	Recovery or survivorship	• Patients decide usage frequency and reminder activation/1 year	Cancer nurse specialists	• Urinary continence (EPIC) [IG: 68.03 (23.25) & CG: 73.62 (23.23)] (NR) • Sexual function (EPIC) [IG: 30.21 (23.67) & CG: 30.32 (24.00)] (NR)	-
Zhang S et al., 2025, China	Assess efficacy of innovative web-based, patient-empowered CRF management program on improving CRF, self-efficacy, and QOL in gastric cancer patients undergoing chemotherapy.	Theory of Unpleasant Symptoms; Conceptual Model of Patient Empowerment; Self-efficacy Theory	Web	• Recognize gastric cancer, overview of fatigue management • Nutrition management • Emotion management • Exercise management • Sleep management • Pain management • Symptom management of nausea and vomiting • Symptom management of diarrhea and altered taste • Online support and telephone follow-up	Undergoing treatment	• Weekly/12 weeks	Nurse	• Fatigue (CFS) [IG: 14.22 (7.39) & CG: 28.34 (9.16)] (*p* = 0.000)	• QOL (EORTCQLQ-C30) [IG: 10.67 (1.26) & CG: 22.97 (2.14)] (*p* = 0.000) • Anxiety (HADS-A) [IG: 2.41 (0.26) & CG: 5.05 (0.48)] (*p =* 0.000) • Depression (HADS-D) [IG: 3.46 (0.38) & CG: 7.63 (0.58)] (*p =* 0.000) • Pain (BPI) [IG: 11.68 (3.87) & CG: 13.88 (3.43)] (*p =* 0.253)
Fjell M et al., 2020, Sweden	Evaluate whether use of interactive app Interaktor improves patients’ levels of symptom burden and HRQOL during neoadjuvant chemotherapy for breast cancer.	NR	App	• Self-reporting of 14 common symptoms	Undergoing treatment	• Daily on weekdays/18 weeks	Nurse	• Symptom severity (MSAS) [IG: 0.80 (NR) & CG: 0.98 (NR)] (*p* = 0.030) • QOL (EORTC QLQ-C30) [IG: 58.21 (NR) & CG: 54.82 (NR)] (*p* = 0.315)	-

**Abbreviations**: Hopkins Symptom Checklist 20-item depression scale, HSCL-20; Brief Pain Inventory, BPI; 12-Item Short-Form Health Survey, SF-12; 7-item Generalized Anxiety Disorder scale, GAD-7; Symptom Representation Questionnaire, SRQ; State-Trait Anxiety Inventory, STAI; 6-item Pain Impact Questionnaire, PIQ-6; Hospital Anxiety and Depression Scale–depression, HADS-D; Hospital Anxiety and Depression Scale–anxiety, HADS-A; Functional Assessment of Chronic Illness Therapy–Spiritual Well-being, FACIT-SP; Quality of Life Questionnaire core 30, QLQ-C30; Memorial Symptom Assessment Scale, MASA; Chinese version of the MD Anderson Symptom Inventory, MDASI-C; Chinese version of the Functional Assessment of Cancer Therapy-Breast, FACT-B; MOS 36-Item Short-Form Health Survey, SF-36; Breast Cancer and Lymphedema Symptom Experience Index, BCLE-SEI; Expanded prostate cancer index composite, EPIC; Cancer Fatigue Scale, CFS; European Organization for Research and Treatment of Cancer Quality of Life Questionnaire, EORTCQLQ-C30; Quality of Life, QOL; Health-Related Quality of Life, HRQOL; Non-Small Cell Lung Cancer, NSCLC; Representational Approach, RA; The-Optimal-Lymph-Flow, TOLF; Cancer-related Fatigue, CRF; Written Representational Intervention to Ease Symptoms, WRITE; WeChat-based cognitive behavioral stress management, WB-CBSM; Cognitive behavioral stress management, CBSM; Information-Motivation-Behavioral Skills, IMB; WeChat-based Education and Rehabilitation Program, WERP; Internet-delivered Mindfulness-Based Cancer Recovery, iMBCR

#### Intervention aims and theoretical basis

Most interventions focused on physical symptom relief, psychological support, or quality of life enhancement, guided by diverse theoretical frameworks grouped into three categories: ① behavioral and motivational theories (e.g., Self-Determination Theory, Self-Efficacy Theory, Information-Motivation-Behavioral Skills Model, and Empowerment Education Model) [23, 24, 32] aimed at promoting health behavior change and engagement; ② symptom and self-management theories (e.g., Theory of Unpleasant Symptoms, Self-Care for Lymphedema Symptom Management Model, Representational Approach, and Patient Empowerment Model) [19, 26, 28, 30] designed to improve symptom understanding and self-care; and ③ psychological and emotional adaptation theories (e.g., Cognitive Behavioral Stress Management, Life Review Theory, Barrera and Castro’s Heuristic Framework) [21, 27, 29] supporting emotional adjustment and coping.

#### mHealth platforms and nurse roles

Nurse-led mHealth interventions employ various digital platforms, with mobile apps (*n* = 4) [20–22, 25, 32] and WeChat (*n* = 3) [21, 27, 29] being the most common. Other platforms included web-based tools (*n* = 2) [19, 28] and a chatbot (*n* = 1) [23]. Multi-channel approaches (*n* = 4) [24, 26, 30, 31] combine formats such as the web, apps, or telephone to increase flexibility and user engagement.

Nurses were the primary providers or coordinators of care in all interventions. Their roles included symptom monitoring, follow-up management, health education, and providing individualized feedback. In several studies, nurses collaborated with physicians, psychologists, or physical therapists as part of multidisciplinary teams.

#### Intervention components and dosage

The core intervention components included symptom monitoring, health education, emotional support, cognitive behavioral strategies, and exercise guidance. The distribution of intervention types by cancer type according to the Omaha System nursing intervention classification [18] is illustrated in Additional file 2: Figure S3. Most studies have focused on case management [20, 23, 27] and teaching, guidance, and counseling [25, 26, 30] interventions for patients with breast cancer. Case management has also been applied in studies involving mixed [31], cervical [21], lung [22], prostate [24], gastric [28], ovarian [19], and colorectal [32] cancers, whereas one study [29] targeted teaching, guidance, and counseling for digestive system cancers. No studies addressed surveillance, treatment, or procedural interventions. The intervention duration ranged from 4 to 24 weeks, with the contact frequency varying from daily to biweekly; some studies have employed both synchronous and asynchronous delivery to increase flexibility and engagement.

#### Assessment of outcomes

Follow-up was categorized as short-term (0–6 months), medium-term (6–12 months), or long-term (≥ 12 months). Most studies (*n* = 9) [19, 20, 23, 25–28, 30, 32] have employed short-term follow-up, ranging from 4 weeks to 5 months. Two studies [21, 29] used medium-term follow-up to assess outcomes up to 6 months. Only three studies [22, 24, 31] implemented long-term follow-up (12 months), enabling the assessment of sustained symptom trajectories and adaptation.

Symptom severity was the most frequently assessed outcome (*n* = 8), typically measured with validated tools such as the MD Anderson Symptom Inventory (MDASI) or the Edmonton Symptom Assessment System (ESAS). Health-related quality of life (HRQOL) was evaluated in seven studies via instruments such as the Functional Assessment of Cancer Therapy-General (FACT-G) and the European Organization for Research and Treatment of Cancer Quality of Life Questionnaire (EORTC QLQ-C30). Psychological outcomes, including anxiety (*n* = 6) and depression (*n* = 5), were commonly assessed with measures such as the Hospital Anxiety and Depression Scale (HADS). Other reported outcomes included physical health, mental health, pain, lymphedema symptoms, fatigue, urinary continence, and sexual function. Further details are provided in Table 3.

### The functional elements, design strategies, use strategies, and evaluation of mHealth

A systematic summary of the functional elements, design and use strategies, and evaluation of the included mHealth interventions was conducted and may be found in Additional file 1: Table S2.

#### 1.1.1. Functional elements

The mHealth interventions provided several core functions: ① symptom assessment, enabling patient self-reporting via mobile platforms (*n* = 4); ② monitoring and early warning, allowing nurses to track data and trigger alerts on the basis of assessments or risk models (*n* = 2); ③ health education through multimedia materials and phone-based instruction (*n* = 12); ④ reminders prompting patients to complete assessments or self-management tasks (*n* = 5); ⑤ consultation and guidance addressing physical, psychological, or rehabilitation concerns (*n* = 6); ⑥ individualized interventions offering tailored goals, self-care plans, and interactive modules based on patient-reported symptoms (*n* = 8); and ⑦ non-individualized interventions providing general self-care advice, psychological or mindfulness support, and rehabilitation exercises (*n* = 5).

#### Design strategies

The mHealth interventions employed various design strategies, including: ① theoretical frameworks (*n* = 10), reflecting diverse behavioral, psychological, and symptom management perspectives, while four studies did not report any explicit theoretical foundation; and ② human-centered interface designs featuring user-friendly layouts (*n* = 1), visual content such as charts, images, and videos *(n* = 6), and health literacy-tailored approaches to enhance comprehension and usability (*n* = 2).

#### 1.1.2. Use strategies

Strategies to promote the adoption and sustained use of mHealth among patients with cancer included the following: ① training and guidance through pre-intervention sessions (*n* = 3) or instruction manuals (*n* = 5) to support independent use; ② professional support via multidisciplinary collaboration among nurses, pharmacists, physicians, and psychologists (*n* = 2), complemented by technical assistance via phone (*n* = 1); and ③ incentives such as free mobile data packages to encourage engagement (*n* = 1).

#### 1.1.3. Evaluation

Among the 14 included studies, 8 reported evaluation data: four assessed feasibility through backend data, patient compliance, and interviews, whereas the other four evaluated usability via scales, interviews, and the think-aloud method. Only one study measured patient satisfaction via a questionnaire.

### Psychological outcomes

#### Significant reduction in mean depression levels

Five studies [21, 22, 28, 29, 31] including 768 patients evaluated the effect of mHealth interventions on depression and reported a significant benefit (SMD = − 1.60, 95% *CI* [− 2.50, − 0.69], *p* < 0.001; Fig. 2a), with high heterogeneity (*I²* = 97%). Sensitivity analysis identified Zhang S et al.’s study [28] as the main source of heterogeneity; after its exclusion, heterogeneity decreased to zero (*I²* = 0%), and a random-effects model revealed a smaller but still significant positive effect of mHealth on depression (SMD = − 0.42, 95% *CI* [− 0.57, − 0.27], *p* < 0.001; Fig. 2b).

**Fig. 2a:**
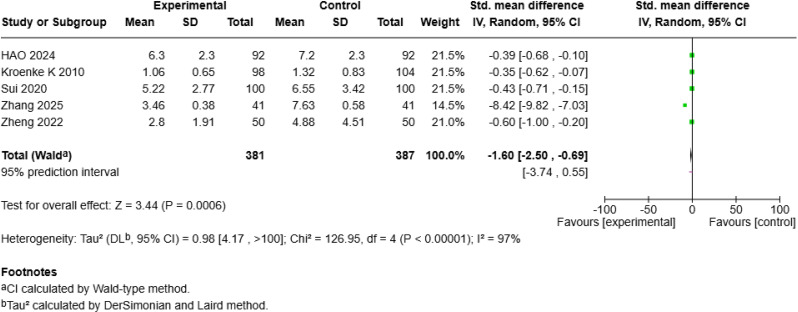
Forest Plot of mHealth Interventions - Depression

**Fig. 2b:**
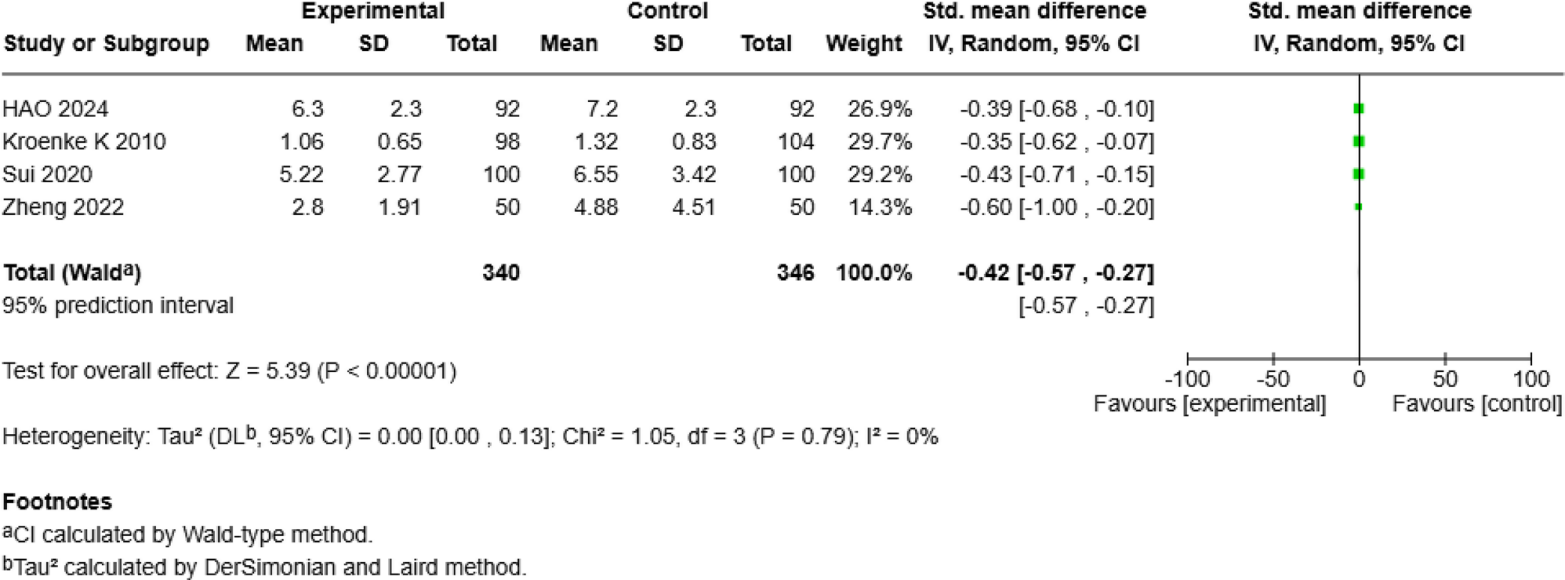
Sensitive Analysis of mHealth Interventions - Depression

**Fig. 2c:**
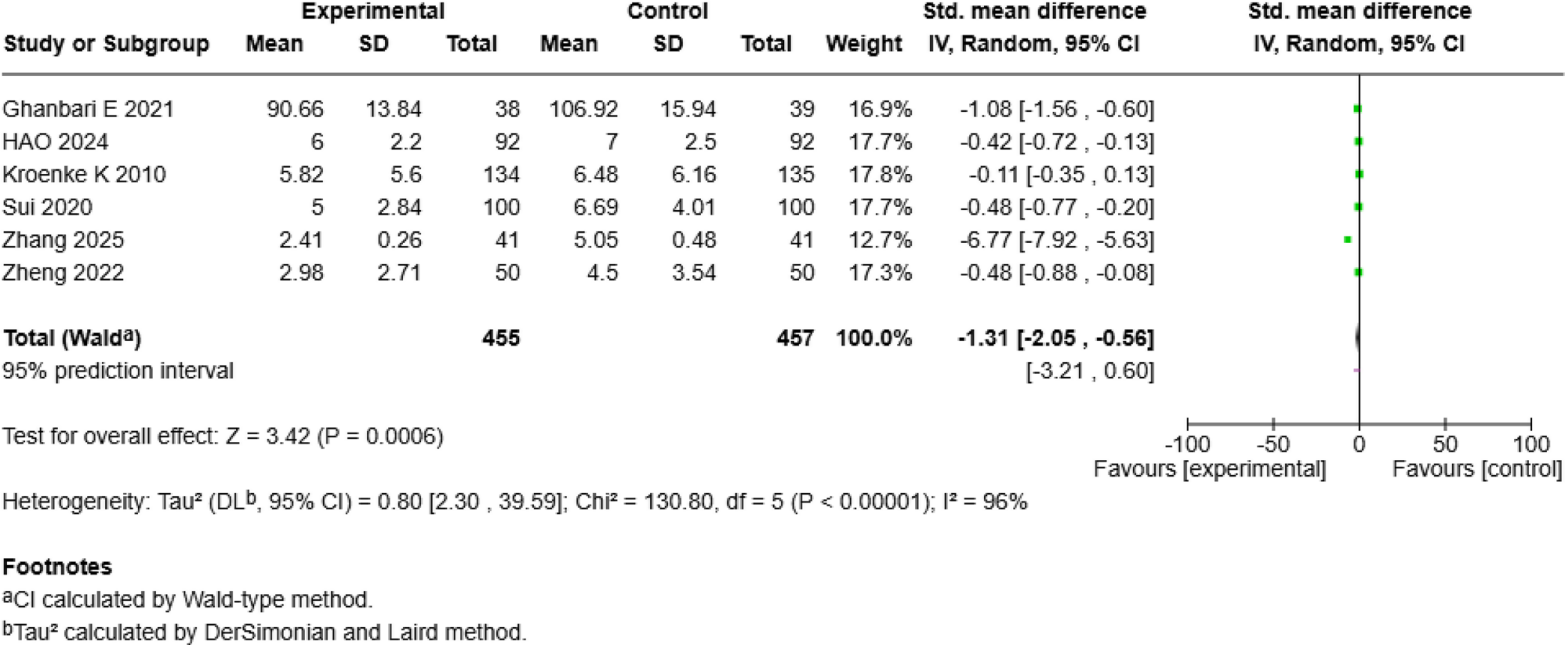
Forest Plot of mHealth Interventions - Anxiety

**Fig. 2d:**
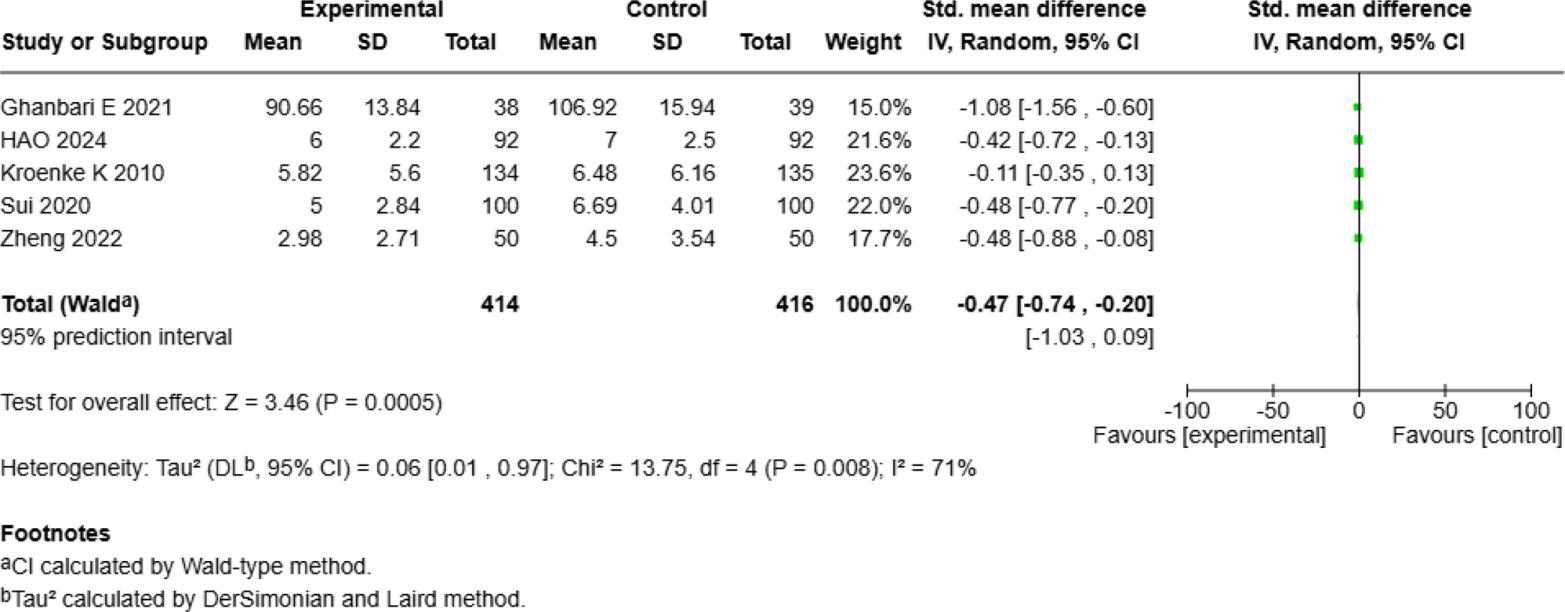
Sensitive Analysis of mHealth Interventions - Anxiety

**Fig. 2e:**
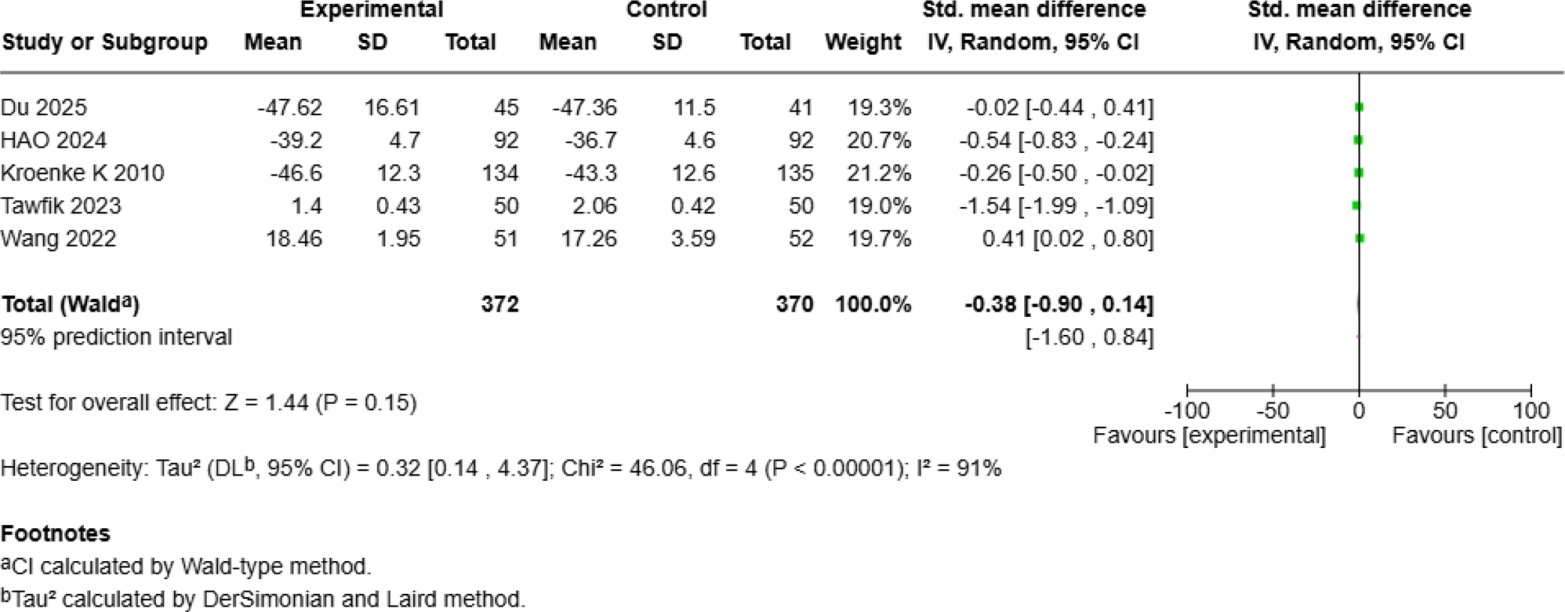
Forest Plot of mHealth Interventions - Mental Health

#### Significant reduction in mean anxiety levels

Six studies [18–19, 22; 25–26, 28] with a total of 912 patients assessed the effect of mHealth interventions on anxiety, revealing a significant reduction (SMD = − 1.31, 95% CI [− 2.05, − 0.56], *p* < 0.001; Fig. 2c) but with high heterogeneity (*I²* = 96%). Sensitivity analysis revealed that excluding Zhang S et al.’s study [28] reduced heterogeneity to moderate levels (*I²* = 71%). According to a random-effects model of the remaining five studies, compared with the control interventions mHealth interventions still significantly improved anxiety outcomes (SMD = − 0.47, 95% *CI* [− 0.74, − 0.20], *p* < 0.001; Fig. 2d).

#### No significant improvement in mental health

Five studies [21, 23, 26, 27, 31] including 742 patients assessed mental health outcomes and reported no significant effects (SMD = − 0.38, 95% *CI* [− 0.90, 0.14], *p* = 0.15; Fig. 2e).

### Physical outcomes

#### Significant reduction in mean symptom severity levels

The meta-analysis of symptom severity included eight studies [19, 21–23, 26, 27, 31, 32] with 1,042 patients with cancer and demonstrated a significant reduction in symptom severity following mHealth interventions (SMD = − 0.91, 95% *CI* [− 1.50, − 0.32], *p* = 0.003; Fig. 3a), with high heterogeneity (*I²* = 95%). Sensitivity analysis revealed that excluding Tawfik E et al.’s study [23] reduced heterogeneity to 81%. According to a random-effects model of the remaining seven studies, compared with control interventions, mHealth interventions still significantly improved symptom severity (SMD = − 0.49, 95% *CI* [− 0.81, − 0.17], *p* = 0.003; Fig. 3b).

**Fig. 3a:**
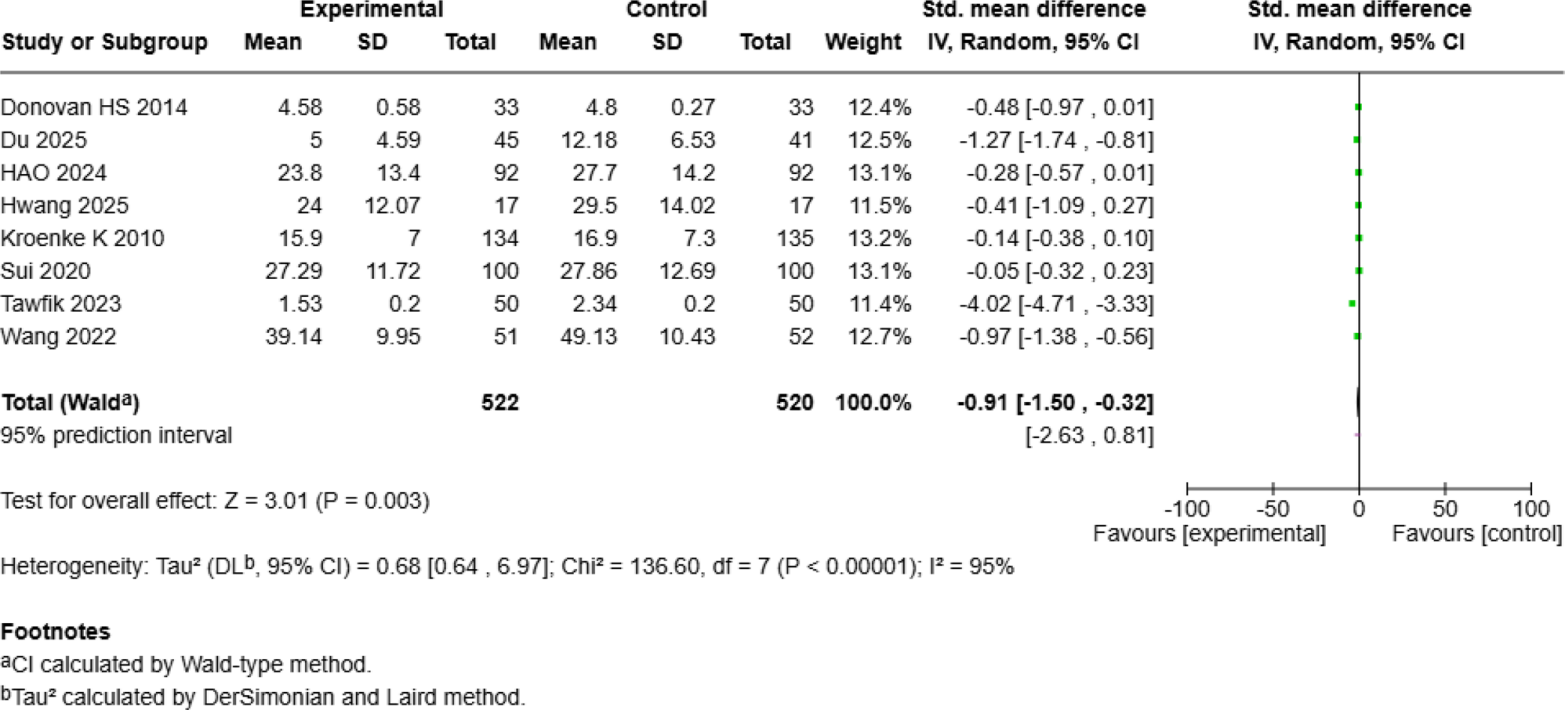
Forest Plot of mHealth Interventions - Symptom Severity

**Fig. 3b:**
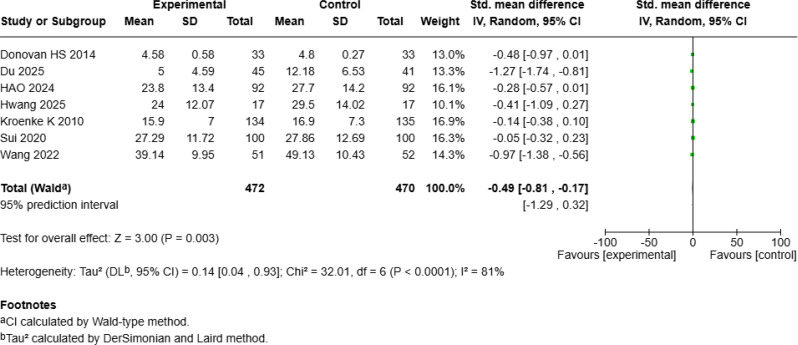
Sensitive Analysis of mHealth Interventions - Symptom Severity

**Fig. 3c:**
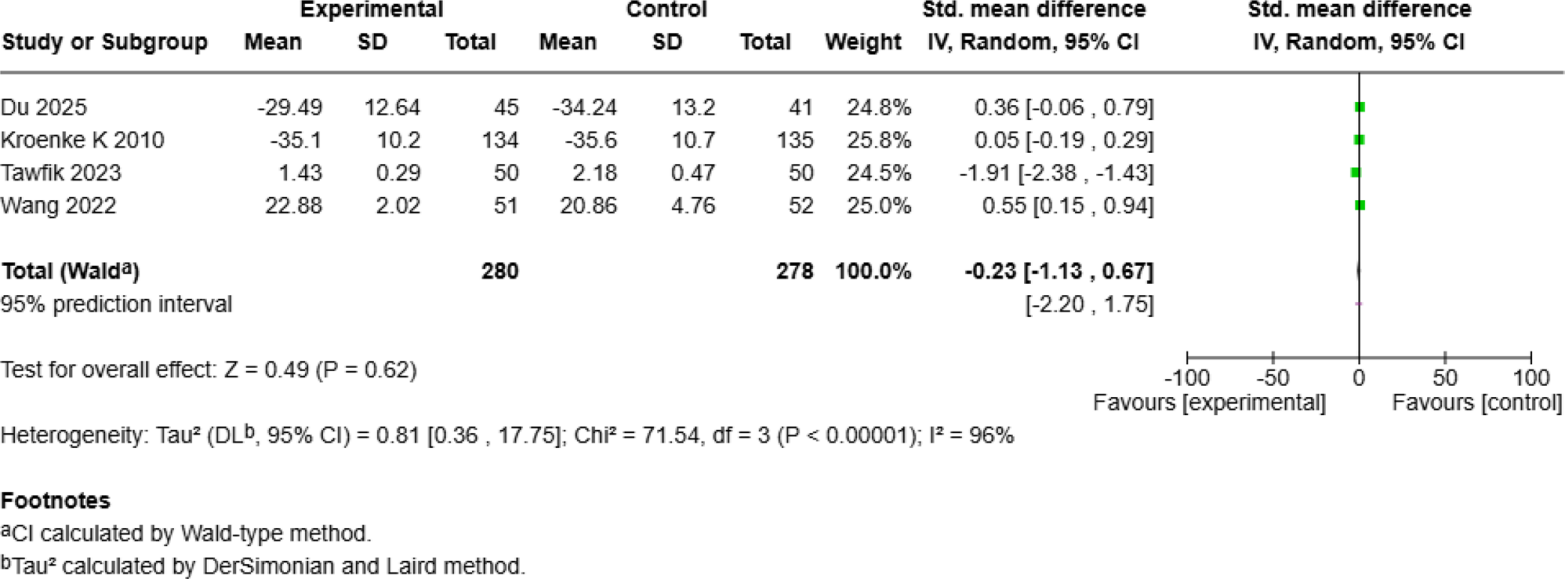
Forest Plot of mHealth Interventions - Physical Health

**Fig. 3d:**
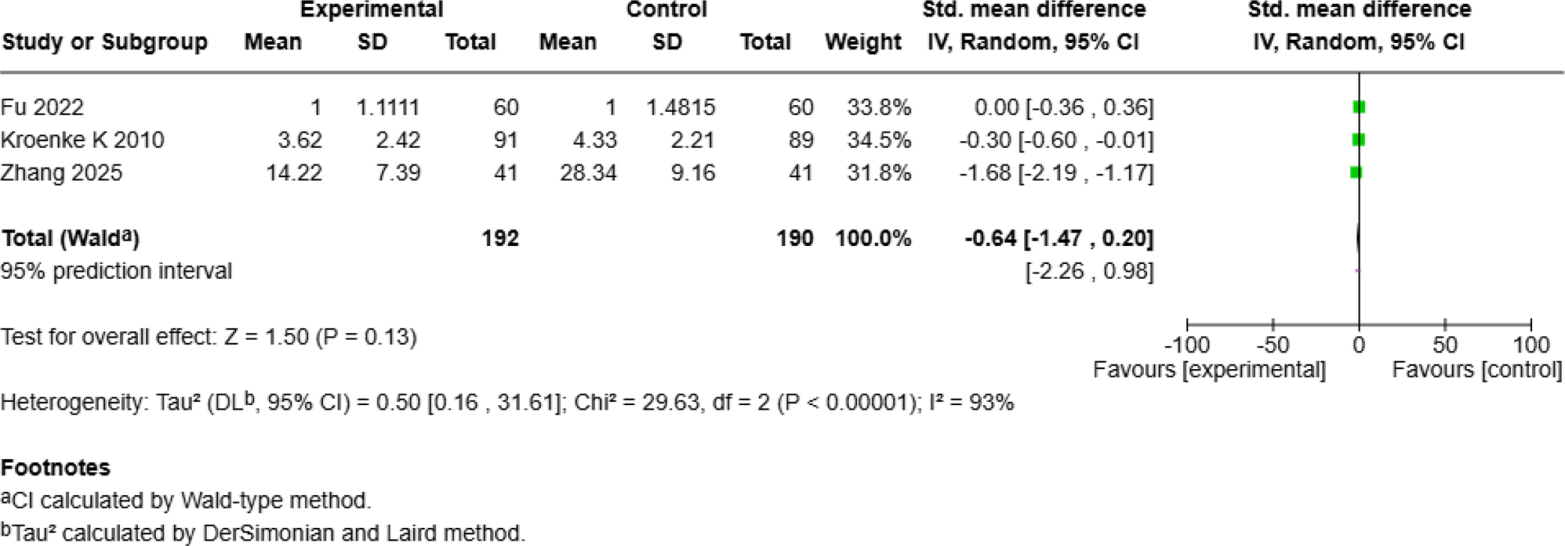
Forest Plot of mHealth Interventions - Pain

#### No significant improvement in physical health or pain

Four studies [23, 26, 27, 31] with 558 patients examined the effect of mHealth interventions on physical health but reported no significant improvement (SMD = − 0.23, 95% *CI* [− 1.13, 0.67], *p* = 0.62; Fig. 3c). Similarly, three studies [28, 30, 31] with 382 patients investigated pain outcomes and reported no significant benefit from mHealth interventions (SMD = − 0.64, 95% *CI* [− 1.47, 0.20], *p* = 0.13; Fig. 3d).

#### Improvement in fatigue in individual studies

One study [28] assessed the efficacy of an innovative web-based, patient-empowered cancer related fatigue (CRF) management program for improving CRF in patients with gastric cancer undergoing chemotherapy. The results revealed that the intervention group had lower physical fatigue scores than did the control group (*p* < 0.001).

#### No significant improvement in lymphedema symptoms, urinary continence or sexual function in individual studies

One study [30] evaluated the effectiveness of the web- and mobile-based TOLF system for managing symptoms related to lymphedema in breast cancer survivors. However, there was no significant difference in the mean number of reported lymphedema symptoms (*p* = 0.11). One study [24] investigated the effects of the ePATH intervention on urinary continence and sexual function. However, no statistically significant differences in urinary continence or sexual function were observed between the intervention and control groups (*p* = 0.09; *p* = 0.97).

### Significant improvement in mean quality of life

The meta-analysis of QOL included seven studies [4, 21, 22, 27, 28, 30, 31] with a total of 992 patients with cancer, revealing a significant positive effect of mHealth interventions (SMD = 1.23, 95% *CI* [0.53, 1.92], *p* < 0.001; Fig. 4a) but with high heterogeneity (*I²* = 96%). Sensitivity analysis revealed that excluding Zhang S et al.’s study [28] reduced heterogeneity to 81%. A random-effects model of the remaining six studies revealed that mHealth interventions continued to significantly improve QOL compared with that of controls (SMD = 0.49, 95% *CI* [0.17, 0.81], *p* = 0.003; Fig. 4b).

**Fig. 4a:**
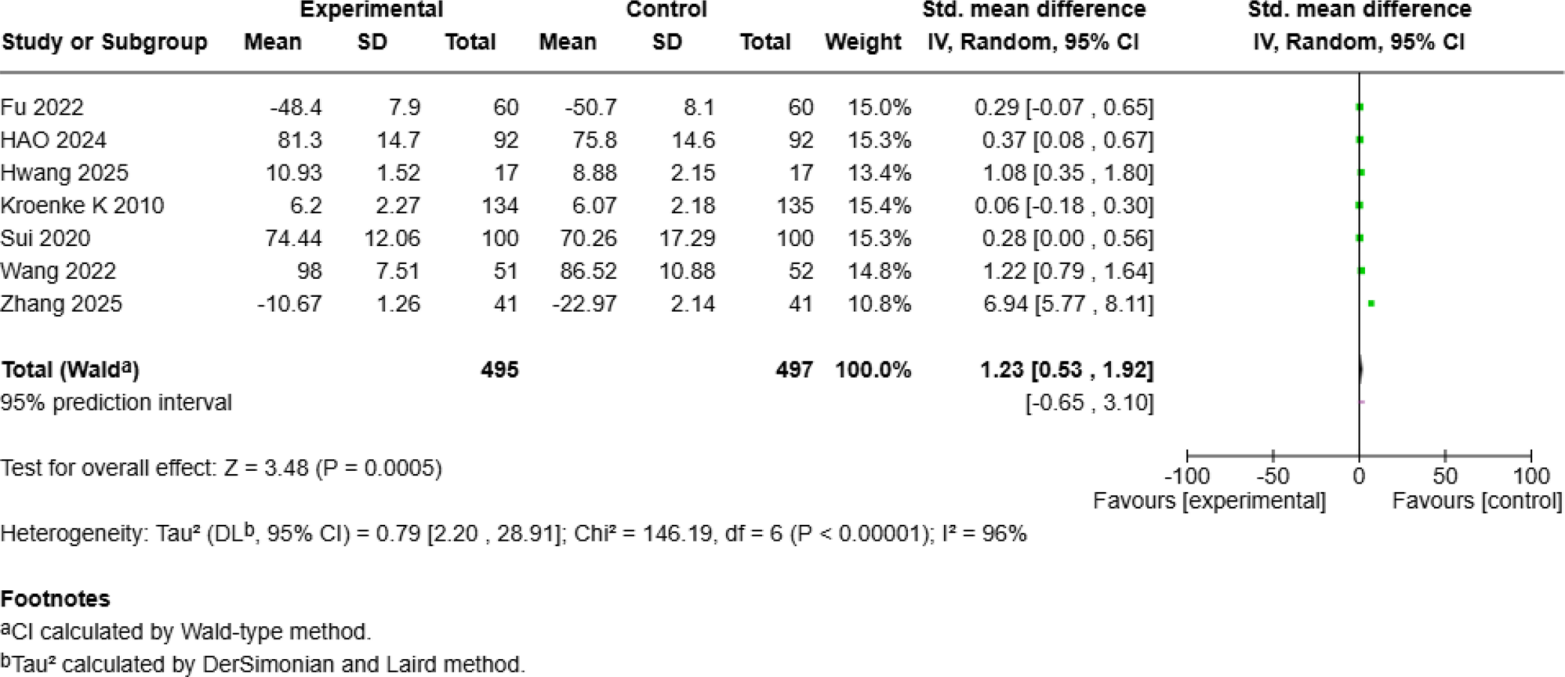
Forest Plot of mHealth Interventions - Quality of Life

**Fig. 4b:**
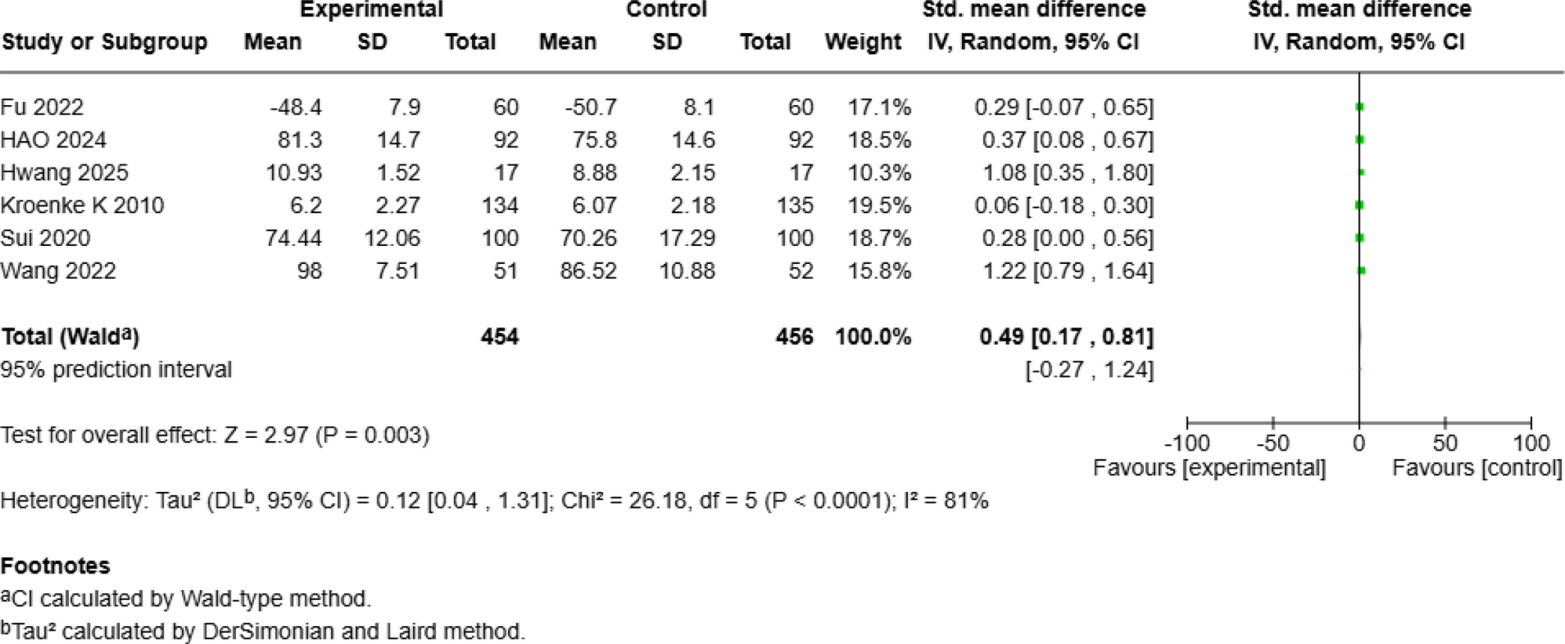
Sensitive Analysis of mHealth Interventions - Quality of Life

### GRADE assessment of evidence quality

The quality of evidence for each outcome included in the meta-analysis was assessed via the GRADE framework. The findings showed that depression was supported by high-quality evidence, whereas QOL was supported by low-quality evidence. In contrast, the outcomes of anxiety, symptom severity, physical health, mental health, and pain were all rated as very low-quality evidence, indicating limited confidence in the effect estimates. The detailed grading results are provided in Additional file 1: Table S3.

### Assessment results based on the mERA checklist

The quality of reporting in the 14 included studies, assessed via the 16-item mERA checklist, was generally suboptimal and inconsistent. Only two studies (14.3%) reported 9 out of 16 items, whereas the majority (85.7%) reported between 3 and 7 items. Frequently addressed domains included intervention content (92.9%), intervention delivery (92.9%), and technology platforms (85.7%). However, key areas such as infrastructure (7.1%), usability/content testing (28.6%), user feedback (42.9%), participant access (7.1%), scalability limitations (14.3%), contextual adaptability (21.4%), replicability (35.7%), and data security (7.1%) have rarely been reported. Notably, none of the studies addressed the interoperability/health information systems (HIS) context, cost assessment, or compliance with national guidelines or regulatory statutes. The detailed results are provided in Additional file 1: Table S4.

## Discussion

Unlike previous reviews, which often overlooked nurse-led interventions [7, 33, 34] or focused narrowly on specific cancer types [13] or treatment modalities [14], this systematic review uniquely centers on nurse-led mHealth interventions for symptom outcomes across diverse cancer populations. The included studies span a wide geographic range, with nearly half conducted in Asia and others from North America, Europe, Africa, and the Middle East. They address a broad spectrum of cancer types, including breast, cervical, colorectal, lung, gastric, prostate, and mixed diagnoses. Our findings highlight the value of these interventions in significantly reducing symptom severity, depression, and anxiety while improving QOL. However, their effects on physical health, pain, and other functional outcomes remain unclear because of limited and mixed evidence. Notably, this is the first review to assess reporting quality via the WHO mERA checklist, offering important insights into the transparency and replicability of nurse-led digital health research in cancer care.

### Refining the design and personalization of nurse-led mHealth interventions

Across the 14 included RCTs, nurse-led mHealth interventions varied considerably in platform, duration, intensity, and theoretical underpinning. Mobile apps and WeChat were the most frequently used platforms, followed by web-based systems. However, few studies have provided rationales for intervention frequency or duration. Most interventions are multi-component, incorporating symptom monitoring, education, emotional support, and self-management, although their structure and emphasis vary. While many were informed by behavioral, psychological, or symptom management theories, none explicitly employed user-centered design principles or digital health–specific frameworks such as the Technology Acceptance Model (TAM) [35, 36] or the Unified Theory of Acceptance and Use of Technology (UTAUT) [37, 38]. This theoretical gap may limit the optimization of mHealth features related to personalization, engagement, and sustained use. Future research should integrate such frameworks to increase the effectiveness and scalability of nurse-led mHealth interventions in cancer care.

Most interventions, classified under the Omaha System, emphasized “teaching, guidance and counseling” and “case management,” particularly for patients with breast cancer. No studies have addressed the “surveillance” or “treatments and procedures” domains, reflecting a narrow focus on education and psychosocial support while underutilizing key nursing roles such as symptom monitoring, physician referrals, and medication management. Given that these interventions are often delivered during active treatment or early rehabilitation—when patients are home and symptoms may evolve rapidly—integrating structured monitoring and timely referrals could enhance responsiveness, prevent complications, and reduce emergency care use. Future mHealth strategies should shift from passive education to active, responsive care to meet the complex and dynamic needs of patients with cancer during home-based recovery.

Another key factor is the level of nurse-patient interaction and personalization. While all interventions were nurse-led, nurse roles varied widely—from real-time feedback and one-on-one communication to automated, generic messaging. Asynchronous feedback and interactive features can strengthen emotional connection, trust, and self-efficacy, which are vital for adherence and behavior change. However, few studies have dynamically tailored content to individual symptom profiles; personalization has been limited mostly to optional modules or general advice. This may partly explain the modest impact on physical and mental health outcomes. Future mHealth interventions should incorporate continuous symptom monitoring, real-time patient-reported outcomes, and nurse-patient communication tools to enable personalized goal setting and feedback. Additionally, integrating machine learning-based risk prediction models could improve responsiveness to evolving symptom needs in home-based cancer care.

### Effectiveness across key outcomes: interpretation and implications

Consistent with previous research [39, 40], this meta-analysis revealed significant reductions in anxiety and depression in the intervention groups, underscoring the effectiveness of nurse-led psychological support. Interventions often incorporate cognitive behavioral techniques, emotion regulation, and stress management, with nurses playing an active, personalized role through one-on-one communication, asynchronous feedback, or group messaging. This continued nurse involvement likely enhanced emotional connection, trust, and continuity of care—key factors in improving psychological outcomes.

Unlike the improvements observed in depression and anxiety, no significant effect was found on general mental health. This may be attributed to the use of broader, less sensitive measures, such as QOL subscales, rather than symptom-specific tools, such as the HADS or Patient Health Questionnaire (PHQ). Moreover, most interventions focused on reducing emotional distress rather than enhancing overall psychological well-being. Given the multidimensional nature of mental health, short-term interventions may be inadequate for meaningful change. Future research should develop interventions that go beyond symptom reduction to promote psychological well-being, use more sensitive and comprehensive assessment tools, and consider longer durations and follow-up to capture sustained effects.

Consistent with previous reviews [41, 42], this study revealed that nurse-led mHealth interventions effectively reduced symptom severity. However, no significant improvement was observed in overall physical health, diverging from some earlier findings [43]. This discrepancy may reflect differences in measurement focus: tools such as the MDASI and ESAS capture symptom intensity, which can respond quickly to interventions promoting awareness, self-monitoring, and emotional support. In contrast, physical health measures (e.g., the SF-36 PCS and EORTC QLQ-C30 physical domain) assess broader domains, such as mobility, energy, and daily functioning, which likely require longer-term or more intensive interventions. Most studies have emphasized symptom management and psychoeducation, with a limited focus on physical rehabilitation. The short intervention durations may also have had a limited impact. Future research should consider integrating structured exercise, nutritional support, or physiotherapy components to better target physical health outcomes.

This review revealed that nurse-led mHealth interventions did not significantly reduce pain intensity, which is consistent with mixed results from prior systematic reviews [44, 45]. The complexity of cancer pain—shaped by disease stage, treatment, and biopsychosocial-spiritual factors—may explain these inconsistencies. While mHealth tools can improve pain awareness and coping, their impact on nociceptive mechanisms is limited without integrated medication management. Notably, Zheng et al. [45] reported that an app with instant messaging communication significantly alleviated cancer pain, unlike one lacking such features. Most interventions targeted general symptoms or psychosocial support rather than pain specifically. Future research should focus on nurse-led, multidisciplinary mHealth interventions that include clinicians, pharmacists, psychotherapists, and rehabilitation specialists. Integrating pain-specific elements—such as symptom monitoring, education, pharmacological support, and rehabilitation—and communication functions may improve the management of cancer-related pain.

Although consistent with the results of previous reviews [46–48], where mHealth interventions significantly improved quality of life, there was still substantial heterogeneity after sensitivity analysis in this study. The evidence suggests that the strongest QOL improvements occurred in interventions incorporating physical activity, cognitive behavioral therapy, or mindfulness [49]. The observed heterogeneity may stem from differences in cancer types, each with unique treatment regimens and recovery needs. For example, exercise and mindfulness may benefit breast cancer survivors dealing with fatigue and distress, whereas patients with lung or gastrointestinal cancers may face complex issues such as respiratory or nutritional challenges, limiting responsiveness to generic interventions. To enhance effectiveness, future mHealth interventions should incorporate cancer type-specific modules tailored to patients’ distinct needs, thereby optimizing QOL outcomes.

### Challenges in evidence quality and reporting transparency

While the meta-analysis revealed promising effects of nurse-led mHealth interventions on several outcomes, the overall certainty and reporting quality of the evidence were limited. According to the GRADE assessment, only depression was rated as high-certainty evidence; QOL was rated low, whereas anxiety, symptom severity, pain, and physical and mental health were rated very low. These ratings were primarily downgraded due to high heterogeneity and imprecision from small sample sizes. As a result, conclusions—especially for anxiety and symptom severity—remain fragile and may change with future research. Persistent heterogeneity in QOL outcomes, even after sensitivity analyses, underscores the need for standardized outcome measures and more tailored interventions across cancer types.

The mERA checklist revealed significant gaps in reporting quality. Most studies do not adequately report key elements such as user feedback, data security, cost assessment, or integration with existing health systems, limiting reproducibility, scalability, and clinical translation [6]. While tools such as CONSORT and STROBE focus on methodological rigor, they overlook technical details critical for understanding and implementing mHealth interventions—gaps that mERA aims to fill. Despite this, many studies fell short of mERA standards, particularly regarding feasibility, user accessibility, and system integration. To strengthen future research, trials should enhance methodological rigor (e.g., proper randomization, sample size calculation, longer follow-up) and adhere to comprehensive reporting frameworks to improve transparency, replication, and real-world adoption of nurse-led mHealth in cancer care.

### Limitations

The studies included in the review have several limitations. First, many of the included studies had notable methodological weaknesses, such as a high risk of bias from inadequate blinding, which may affect the reliability of their findings. Second, most studies had relatively short follow-up periods, limiting insight into long-term intervention effects. Third, the majority of studies were conducted in urban settings, raising concerns about the generalizability of mHealth interventions to underserved or rural populations with limited healthcare access. Finally, poor reporting quality in many studies has hindered comprehensive synthesis and replication.

This review has several limitations. Restricting inclusion to published literature may introduce publication bias since more statistically significant or positive outcomes are more likely to be published than studies with negative, nonsignificant, or inconclusive findings, thus yielding a potential overestimate of the true effect of interventions. Despite comprehensive searches, non-English and non-Chinese studies were excluded, potentially omitting relevant data. Because fewer than ten studies contributed to each outcome, statistical tests such as Egger’s regression or funnel plot asymmetry would be underpowered and potentially misleading. Nonetheless, publication bias cannot be excluded. Trials with nonsignificant or unfavorable results may be less likely to be published, which could lead to an overestimation of intervention effects. The risk of bias and GRADE assessments involve subjective judgments, which could cause inter-rater variability. Some studies were excluded from meta-analyses because of missing data or high bias risk, limiting the evidence base. The small number of included studies prevented subgroup analyses by cancer type or intervention duration, restricting exploration of heterogeneity. Additionally, the limited number of eligible studies and incomplete outcome data meant that several outcomes were synthesized narratively rather than quantitatively, possibly contributing to non-significant physiological findings. The outcomes reported by only two or three studies should be interpreted cautiously given their uncertain reliability and generalizability. Finally, the Wald-type method may underestimate the uncertainty when the number of trials is small, whereas the Hartung-Knapp adjustment may provide more conservative intervals. We therefore recommend that the results be interpreted with caution.

### Implications for practice and research

This review offers key implications for future research and practice. Future studies should improve the methodological rigor with proper randomization, larger samples, longer follow-ups, and standardized validated outcomes. Interventions must be theory-driven and tailored to specific cancer types to address unique symptom profiles. To reduce reporting gaps, adherence to mobile health standards such as mERA is essential for transparency and comparability. Incorporating a user-centered design and evaluating usability will enhance real-world applicability. Although nurse-led mHealth interventions significantly improved psychological outcomes, such as depression and anxiety, the magnitude of the effect may be modest from a clinical perspective. For example, the pooled effect on depression (SMD = − 0.42) corresponds to an approximate reduction of 1.1 points on the HADS-D scale (0–21), which is close to but does not fully reach the commonly reported minimal clinically important difference (MCID) of 1.5–1.7 points. In contrast, evidence for the management of physical symptoms remains limited. Given the significant physiological burden in patients with cancer, more research should focus on physical symptom interventions. Current reliance on self-report questionnaires risks recall bias and limited sensitivity; integrating multimodal data collection tools—such as wearables, sensors, and ecological momentary assessment—can provide objective, real-time insights to improve symptom tracking and treatment responsiveness.

Second, from a clinical perspective, nurse-led mHealth interventions show strong potential as scalable and accessible tools for symptom management, particularly in underserved and remote areas with limited healthcare resources. By integrating regional medical alliances—such as collaborations between tertiary hospitals and primary care institutions, including township and community health centers—mHealth platforms may help extend specialized nursing services to peripheral settings, thereby alleviating disparities in care caused by geographic inequity. This model can support the decentralization and equalization of nursing services, ultimately improving symptom management quality among rural and vulnerable populations. However, successful integration requires addressing challenges related to digital literacy, patient engagement, and data security. Nurses’ digital competencies, communication skills, and interdisciplinary collaboration abilities must also be strengthened through structured training and institutional support to ensure that such interventions are effectively embedded into existing care pathways and multidisciplinary frameworks.

## Conclusions

Nurse-led mHealth interventions show promising effects in improving symptom severity, depression, anxiety, and quality of life among patients with cancer, highlighting nurses’ unique role in delivering accessible, supportive, patient-centered digital care. However, their full potential is limited by insufficient personalization, poor reporting transparency, and the underuse of key nursing functions such as symptom surveillance and care coordination. Future research should adopt rigorous methodologies, follow standardized reporting frameworks, tailor interventions to specific cancer types and symptom profiles, and integrate mHealth into multidisciplinary clinical workflows to increase scalability and sustainability.

## Supplementary Information

Below is the link to the electronic supplementary material.

Supplementary Material 1

Supplementary Material 2

## Data Availability

The datasets used and analyzed during the current study are available from the corresponding author upon reasonable request.

## Abbreviations

mHealth: Mobile Health
RCT: randomized controlled trial
SMD: standardized mean difference
CI: Confidence interval
mERA: mHealth Evidence Reporting and Assessment
PROSPERO: International Prospective Register of Systematic Reviews
QOL: quality of life
WHO: World Health Organization
RoB: Risk of Bias
GRADE: Grading of Recommendations Assessment, Development and Evaluation
CFR: cancer related fatigue
TAM: Technology Acceptance Model
UTAUT: Unified Theory of Acceptance and Use of Technology
MDASI: MD Anderson Symptom Inventory
ESAS: Edmonton Symptom Assessment System
FACT-G: Functional Assessment of Cancer Therapy-General
EORTC QLQ-C30: European Organization for Research and Treatment of Cancer Quality of Life Questionnaire
HADS: Hospital Anxiety and Depression Scale
HIS: Health information systems
PHQ: Patient Health Questionnaire
