# Supplementary material for: Effectiveness of nurse-led mHealth interventions on symptom outcomes in adult patients with cancer: a systematic review and meta-analysis
Source: BMC Nurs. 2025 Oct 31;24:1356. doi: 10.1186/s12912-025-03981-2 (PMC12577444; doi:10.1186/s12912-025-03981-2)
Supplement: Supplementary file 1 — Supplementary Material 1 [file 12912_2025_3981_MOESM1_ESM.docx]

**Table S1. Search Strategies**

| **Search Item** | **Category** | **Search Strategy** |
| --- | --- | --- |
| **Cancer** | #1 | "neoplasms"[MeSH Terms] OR "neoplas*"[Title/Abstract] OR "cancer*"[Title/Abstract] OR "carcinoma*"[Title/Abstract] OR "tumo*"[Title/Abstract] OR "malignan*"[Title/Abstract] |
| **Symptom** | #2 | "syndrome"[MeSH Terms] OR "symptom*"[Title/Abstract] OR "symptom cluster*"[Title/Abstract] OR "multiple symptom*"[Title/Abstract] OR "symptom constellation*"[Title/Abstract] OR "concurrent symptom*"[Title/Abstract] OR "co-occurring symptom*"[Title/Abstract] |
| **Mobile health** | #3 | "telemedicine"[MeSH Terms] OR "telecare*"[Title/Abstract] OR "mobile health"[Title/Abstract] OR "mhealth"[Title/Abstract] OR "m-health"[Title/Abstract] OR "ehealth"[Title/Abstract] OR "e-health"[Title/Abstract] OR "telehealth"[Title/Abstract] OR "mcare"[Title/Abstract] OR "m-care"[Title/Abstract] |
|  | #4 | "mobile applications"[MeSH Terms] OR "mobile app*"[Title/Abstract] OR "mobile technology"[Title/Abstract] OR "app"[Title/Abstract] OR "apps"[Title/Abstract] OR "portable electronic app*"[Title/Abstract] OR "portable software app*"[Title/Abstract] OR "smartphone app*"[Title/Abstract] |
|  | #5 | "cell Phone"[MeSH Terms] OR "cell* phone*"[Title/Abstract] OR "smart phone*"[Title/Abstract] OR "mobile phone*"[Title/Abstract] |
|  | #6 | "wearable electronic devices"[MeSH Terms] OR "wearable devices"[Title/Abstract] OR "wearable technology"[Title/Abstract] |
|  | #7 | "software"[MeSH Terms] OR "wechat"[Title/Abstract] OR "small program"[Title/Abstract] OR "mini program"[Title/Abstract] OR "applet"[Title/Abstract] |
|  | #8 | #3 OR #4 OR #5 OR #6 OR #7 |
|  | #9 | #1 AND #2 AND#8 |

**Table S2. Summary of Functional Elements, Design and Use Strategies, and Evaluation in mHealth Interventions**

| **Name of Study or Intervention** | **Author, Year, Country** | **Functional Element** | **Design Strategy** | **Use Strategy** | **Evaluation** |
| --- | --- | --- | --- | --- | --- |
| Indiana Cancer Pain and Depression (INCPAD) trial | Kroenke K et al., 2010, USA | (1); (2); (3); (6) | NM | (2)a | NM |
| WRITE Symptoms | Donovan HS et al., 2014, USA | (1); (3); (6) | (1) | (1)b; (2)a | (2)a; (3) |
| Breast Cancer Support zone (BCS zone) | Ghanbari E et al., 2021, Iran | (3);(7) | (1); (2)b | (1)b; (2)b; (3) | (3) |
| The-Optimal-Lymph-Flow health IT system (TOLF) | Fu MR et al., 2022, USA | (1); (3); (4); (7) | (1); (2)b | NM | (2)a b c; |
| WeChat-Based Cognitive Behavioural Stress Management (WB-CBSM) | Hao Q et al., 2024, China | (3); (5); (6) | (2)c | NM | NM |
| App-Based Physical Activity Program | Hwang YJ et al., 2025, South Korea | (3); (4); (5) | (1); (2)a c; | (1)a | (1)b; |
| WeChat-based Education Rehabilitation Program (WERP) | Sui Y et al., 2020, China | (3); (4); (5); (6) | (2)b; | (1)b | NM |
| ChemoFreeBot | Tawfik E et al., 2023, Egypt | (3); (6) | NM | NM | (2)a |
| internet-delivered Mindfulness-Based Cancer Recovery (iMBCR) program | Wang L et al., 2022, China | (7) | NM | (1)a | (1)b |
| WeChat-based Life Review Program | Zheng M et al., 2022, China | (6); (10) | (1); (2)b | (1)b | NM |
| The-Optimal-Lymph-Flow (TOLF) | Du X et al., 2025, China | (3); (5); (7) | (1); (2)b | (1)a | NM |
| electronic Patient Activation in Treatment at Home (ePATH) | Wennerberg C et al., 2023, Sweden | (3); (4); (5); (6) | (1); | NM | (1)c; (2)c |
| Cancer-Related Fatigue Management Program | Zhang S et al., 2025, China | (3); (5); (6); (7) | (1) | NM | NM |
| Interaktor | Fjell M et al., 2020, Sweden | (1); (2); (3); (4) | (2)b | (1)b | (1)c |

NM, Not Mentioned.

Functional elements: (1) Symptom assessment; (2) Monitoring and early warning; (3) Health education; (4) Remind; (5) Consultation and guidance; (6) Individualized intervention; and (7) Non-individualized intervention.

Design strategies: (1) Theoretical guidance frameworks; and (2) Human-centered interface design: a. User-friendly interface design, b. Visualization, c. Health literacy-based design.

Use strategies: (1) Improving the ability of people with cancer: a. Use training, b. Instruction manuals; (2) Professional support: a. Multidisciplinary cooperation, b. Technical professional support; and (3) Package of free mobile data.

Evaluation: (1) Feasibility: a. Use data, b. Compliance, c. Interviews; (2) Usability: a. Scale, b. Interviews, c. Think aloud technique; and (3) Satisfaction: questionnaire.

**Table S3. Grading of Recommendations Assessment, Development, and Evaluation (GRADE) Evidence Profile**

| Outcome | Risk of Bias^a^ | Inconsistency^b^ | Indirectness^c^ | Imprecision^d^ | Publication Bias^e^ | Certainty of the  Evidence (GRADE)^f^ |
| --- | --- | --- | --- | --- | --- | --- |
| Depression | Not serious | Not serious | Not serious | Not serious | Undetected | ⨁⨁⨁⨁  High |
| Anxiety | Not serious | Very serious | Not serious | Serious | Undetected | ⨁㊀㊀㊀  Very low |
| Quality of life | Not serious | Very serious | Not serious | Not serious | Undetected | ⨁⨁㊀㊀  Low |
| Symptom severity | Serious | Very serious | Not serious | Not serious | Undetected | ⨁㊀㊀㊀  Very low |
| Physical health | Not serious | Very serious | Not serious | Serious | Undetected | ⨁㊀㊀㊀  Very low |
| Mental health | Serious | Very serious | Not serious | Serious | Undetected | ⨁㊀㊀㊀  Very low |
| Pain | Not serious | Very serious | Not serious | Very serious | Undetected | ⨁㊀㊀㊀  Very low |

Note.

^a^ Risk of Bias: when more than two-thirds of included RCTs in each outcome came from studies with some concern, the certainty of evidence was downgraded by one level (-1). When more than two-thirds came from high-risk studies, the certainty was downgraded by two levels (-2).

^b^ Inconsistency: based on the variability and heterogeneity across individual trials. When 50% < *I^2^* < 75%, the certainty of evidence was downgraded by one level (-1); when *I^2^* ≥75%, it was downgraded by two levels (-2).

^c^ Indirectness: assessed qualitatively by extent that population, interventions, and outcome measures directly reflected aims of systematic review.

^d^ Imprecision: based on the inspection of pooled estimate and 95% confidence interval (95% CI). Grade was decreased by one (-1) when the analysis included fewer than 400 participants or if there were wide CIs, and by two (-2) when the number of participants included in analysis was very low or if CIs were very wide.

^e^ Publication bias: assessed by funnel plot and an extension to Egger’s regression test. The certainty downgraded when *p* <0.05.

^f^ High certainty: very confident that the true effect lies close to that of the estimate of effect; Moderate certainty: moderately confident in the effect estimate and the true effect is likely to be close to the estimate of the effect but possibly that it is substantially different; Low certainty: confidence in the effect estimate is limited and the true effect may be substantially different from the estimate of the effect; Very low certainty: very little confidence in the effect estimate and the true effect is likely to be substantially different from the estimate of the effect.

**Table S4. Quality Assessment of mHealth Evidence Reporting using mERA Guidelines**

| **Study** | **1. Infrastructure**  **(Population Level)** | **2.**  **Technology Platform** | **3.**  **Interoperability/**  **HIS Context** | **4.**  **Intervention Delivery** | **5.**  **Intervention Content** | **6.**  **Usability/Content**  **Testing** | **7.**  **User Feedback** | **8.**  **Access of Individual**  **Participants** | **9.**  **Cost Assessment** | **10.**  **Adoption Inputs/**  **Program Entry** | **11.**  **Limitations for**  **Delivery at Scale** | **12.**  **Contextual**  **Adaptability** | **13.**  **Replicability** | **14.**  **Data Security** | **15.**  **Compliance with**  **National Guidelines**  **or Regulatory Statutes** | **16.**  **Fidelity of**  **Intervention** | **Proportion (Reports / Total)** |
| --- | --- | --- | --- | --- | --- | --- | --- | --- | --- | --- | --- | --- | --- | --- | --- | --- | --- |
| Kroenke K et al., 2010 | － | ＋ | － | ＋ | ＋ | － | － | － | － | － | ＋ | ＋ | － | － | － | － | 5/16 |
| Donovan HS et al., 2014 | － | ＋ | － | ＋ | ＋ | ? | ＋ | ? | － | ＋ | － | － | － | － | － | ＋ | 6/16 |
| Ghanbari E et al., 2021 | － | ＋ | － | ? | － | － | ＋ | － | － | ＋ | － | － | － | － | － | － | 3/16 |
| Fu MR et al., 2022 | － | ＋ | － | ＋ | ＋ | ＋ | ＋ | － | － | － | － | － | ＋ | － | － | ＋ | 7/16 |
| Hao Q et al., 2024 | － | ＋ | － | ＋ | ＋ | － | － | － | － | － | － | － | － | － | － | － | 3/16 |
| Hwang YJ et al., 2025 | ＋ | ＋ | － | ＋ | ＋ | － | ? | － | － | ＋ | － | － | － | － | － | ＋ | 6/16 |
| Sui Y et al., 2020 | － | ＋ | － | ＋ | ＋ | － | ＋ | ＋ | － | ＋ | ＋ | － | ＋ | － | － | ＋ | 9/16 |
| Tawfik E et al., 2023 | － | ＋ | － | ＋ | ＋ | ＋ | ＋ | － | － | ＋ | － | ＋ | － | － | － | － | 7/16 |
| Wang L et al., 2022 | － | ＋ | － | ＋ | ＋ | ＋ | ＋ | － | － | ＋ | － | ＋ | ＋ | － | － | ＋ | 9/16 |
| Zheng M et al., 2022 | － | ＋ | － | ＋ | ＋ | － | － | － | － | － | － | － | ＋ | － | － | － | 4/16 |
| Du X et al., 2025 | － | ＋ | － | ＋ | ＋ | － | － | － | － | － | － | － | － | － | － | ＋ | 4/16 |
| Wennerberg C et al., 2023 | － | ＋ | － | ＋ | ＋ | ＋ | ＋ | － | － | ＋ | ＋ | － | ＋ | － | ＋ | ＋ | 4/10 |
| Zhang S et al., 2025 | － | － | － | ＋ | ＋ | － | － | － | － | ＋ | － | － | － | － | － | ＋ | 4/10 |
| Fjell M et al., 2020 | － | － | － | ＋ | ＋ | ＋ | － | － | － | ＋ | － | － | ＋ | ＋ | － | ＋ | 7/10 |
| **Proportion (Reports / Total)** | 1/14 | 12/14 | 0/14 | 13/14 | 13/14 | 4/14 | 6/14 | 1/14 | 0/14 | 8/14 | 2/14 | 3/14 | 5/14 | 1/14 | 0/14 | 9/14 | - |

Note: “－” Indicates that the item was not addressed in the study. “?” Indicates that it is partially reported, and “+” indicates that it is reported.
