# Supplementary material for: Effectiveness of nurse-led mHealth interventions on symptom outcomes in adult patients with cancer: a systematic review and meta-analysis
Source: BMC Nurs. 2025 Oct 31;24:1356. doi: 10.1186/s12912-025-03981-2 (PMC12577444; doi:10.1186/s12912-025-03981-2)
Supplement: Supplementary file 2 — Supplementary Material 2 [file 12912_2025_3981_MOESM2_ESM.docx]

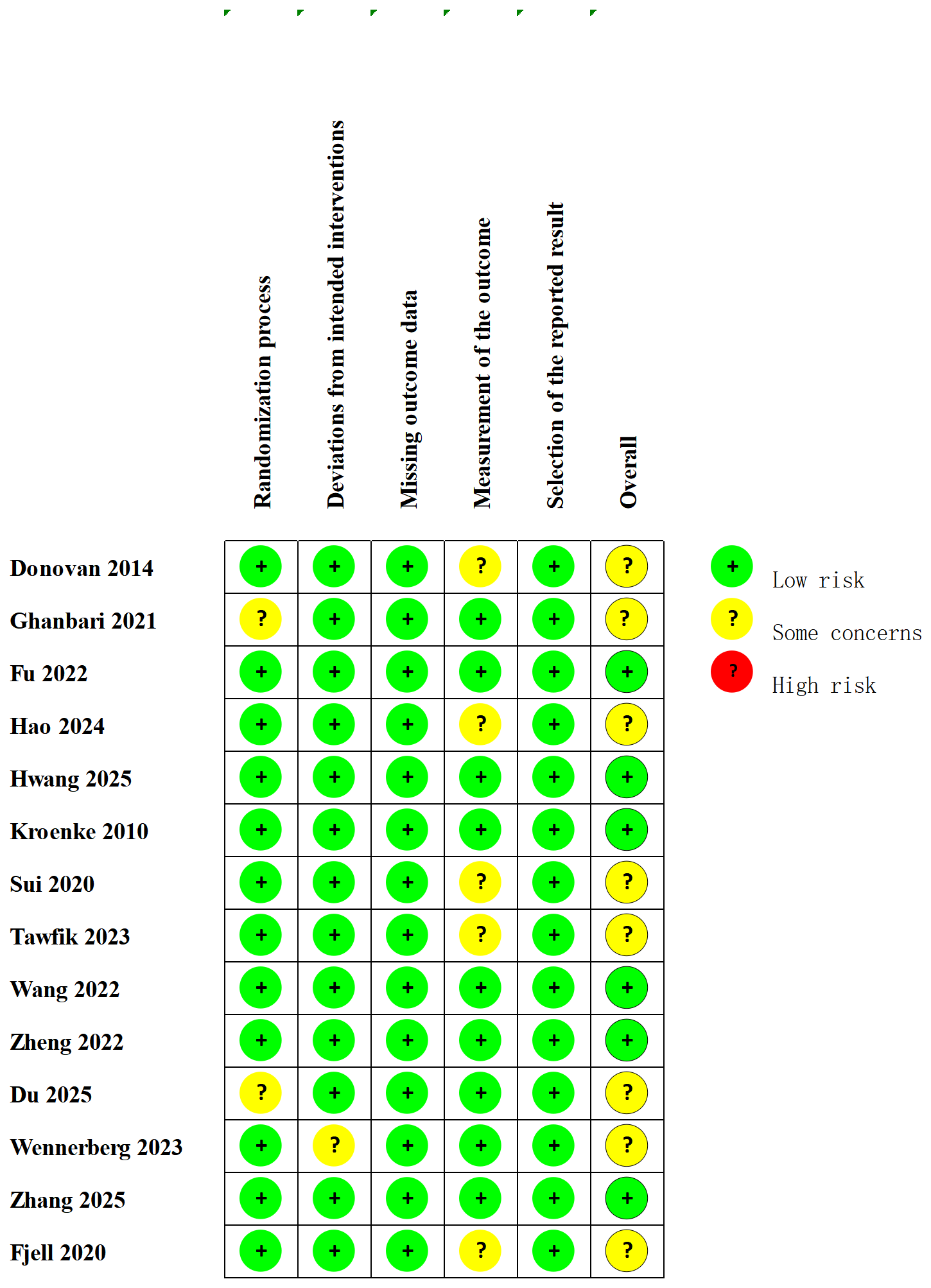


**Figure S1. Risk of Bias Summary**

Note. Green represents a low risk of bias, yellow a moderate risk, and red a high risk.


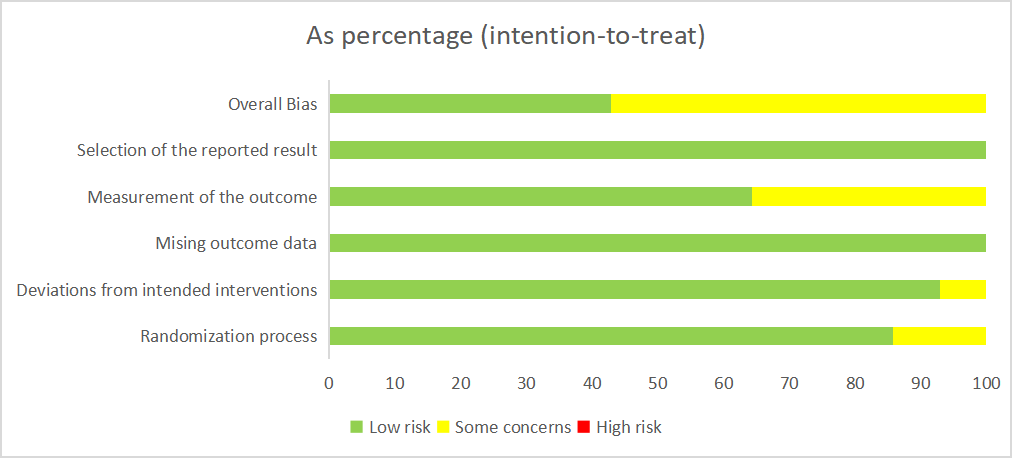


**Figure S2. Graphical Representation of the Risk of Bias Summary by Risk Category**


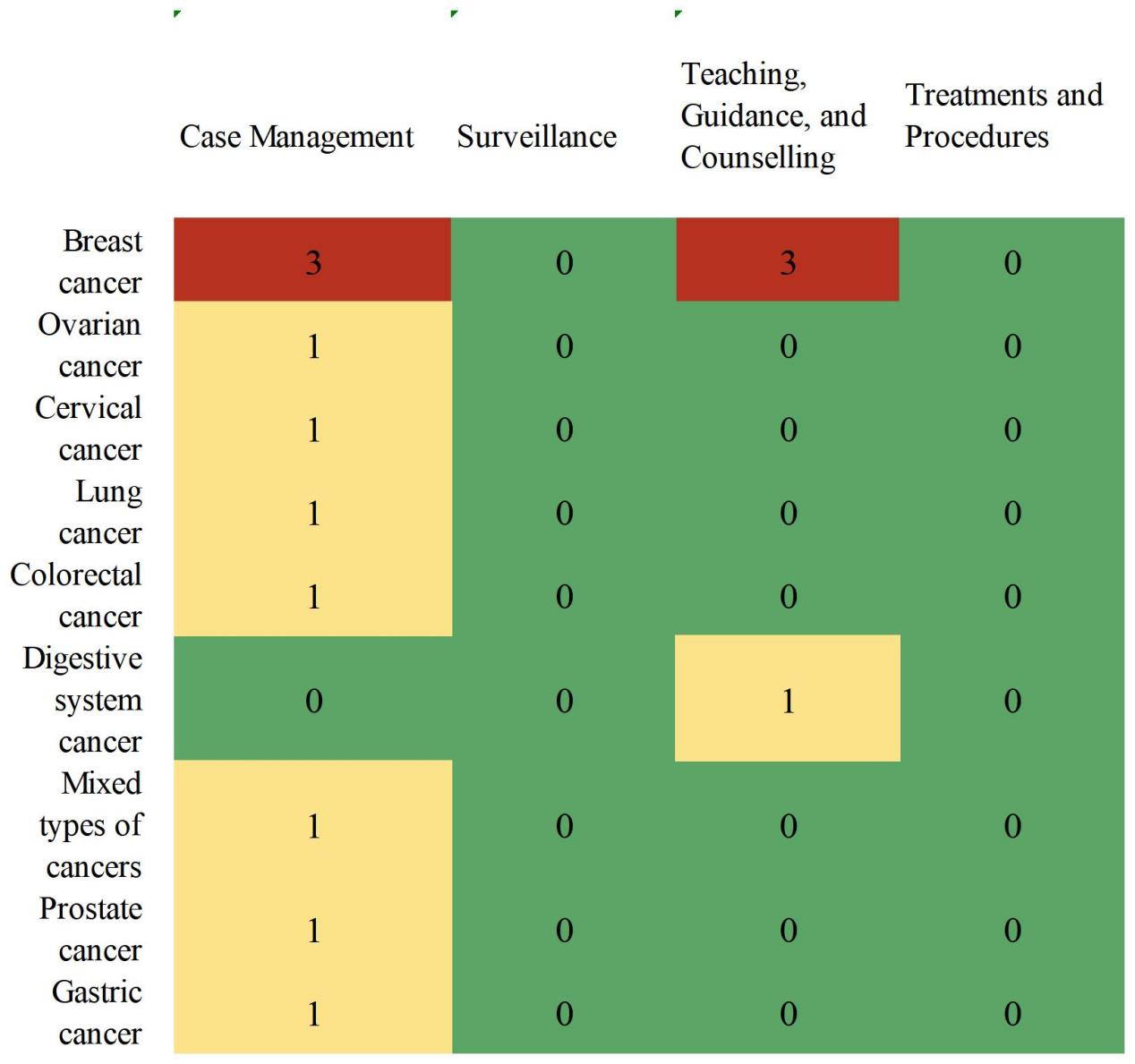


**Figure S3. Distribution of the OMAHA Heat Map Categories Across all Included Studies (n=14) by Cancer Type**

Note: Values with the highest frequency were assigned a red color, middle values a yellow color and lowest values a green color
